# Defective humoral immunity disrupts bile acid homeostasis which promotes inflammatory disease of the small bowel

**DOI:** 10.1038/s41467-022-28126-w

**Published:** 2022-01-26

**Authors:** Ahmed Dawood Mohammed, Zahraa Mohammed, Mary M. Roland, Ioulia Chatzistamou, Amy Jolly, Lillian M. Schoettmer, Mireya Arroyo, Khadija Kakar, Yuan Tian, Andrew Patterson, Mitzi Nagarkatti, Prakash Nagarkatti, Jason L. Kubinak

**Affiliations:** 1 University of South Carolina School of Medicine Department of Pathology, Microbiology, Immunology 6439 Garners Ferry Rd., Columbia, SC 29209 USA; 2 University of Baghdad School of Veterinary Medicine, Baghdad, Iraq; 3 Al-Mustansiriyah University School of Medicine Department of Microbiology, Baghdad, Iraq; 4 Pennsylvania State University Department of Veterinary and Biomedical Sciences, State College, PA USA

**Keywords:** Antimicrobial responses, Immunological disorders, Mucosal immunology, Microbiome, Symbiosis

## Abstract

Mucosal antibodies maintain gut homeostasis by promoting spatial segregation between host tissues and luminal microbes. Whether and how mucosal antibody responses influence gut health through modulation of microbiota composition is unclear. Here, we use a CD19^−/−^ mouse model of antibody-deficiency to demonstrate that a relationship exists between dysbiosis, defects in bile acid homeostasis, and gluten-sensitive enteropathy of the small intestine. The gluten-sensitive small intestine enteropathy that develops in CD19^−/−^ mice is associated with alterations to luminal bile acid composition in the SI, marked by significant reductions in the abundance of conjugated bile acids. Manipulation of bile acid availability, adoptive transfer of functional B cells, and ablation of bacterial *bile salt hydrolase* activity all influence the severity of small intestine enteropathy in CD19^−/−^ mice. Collectively, results from our experiments support a model whereby mucosal humoral immune responses limit inflammatory disease of the small bowel by regulating bacterial BA metabolism.

## Abstract (web summary)

Mucosal antibodies maintain gut homeostasis, and may influence gut health through modulation of microbiota composition. Here the authors use a CD19-deficient mouse model with deficient *B*-*cell immune responses* to uncover an association between humoral immunodeficiency, dysbiosis, and perturbations to bile acid homeostasis in the gut in the context of glute-sensitive enteropathy.

## Introduction

Antibody-deficiency is the most common form of primary (i.e., heritable) immunodeficiency observed in humans, and gastrointestinal complications are commonly observed in antibody-deficient patients^1–5^. Mouse models have been instrumental in demonstrating that mucosal antibodies (particularly immunoglobulin A) regulate the taxonomic composition^6,7^, metabolic output^8^, and spatial organization^9,10^ of the gut microbiota. In antibody-deficient humans, the abnormal composition of the gut microbiota (‘dysbiosis’) is associated with chronic inflammatory responses^7,11–13^. The underlying mechanisms through which dysbiosis causes chronic inflammation are not well-defined.

Under normal physiological conditions, bacterial metabolism in the gut has a profoundly positive influence on host physiology by promoting detoxification of harmful dietary compounds, limiting inflammatory immune responses, and enhancing the bioavailability of nutrients^8,14,15^. One critical metabolic function carried out exclusively by bacteria in the gut is bio-transformation of host bile acids (BAs)^16–18^. BAs are a diverse family of small amphipathic molecules that, through their detergent properties, promote the solubilization and absorption of dietary lipids and lipid-soluble vitamins from the gut lumen^19^. BAs also serve as important signaling molecules that regulate their own synthesis, and influence intestinal epithelial cell (IEC) health and immune cell function^20–25^. BAs are categorized into two types: primary and secondary BAs. Primary BAs refer to host-derived BAs synthesized in the liver and stored in the gall bladder until they are secreted into the duodenum. Secondary BAs are derived from bacterial metabolism of primary BAs, and this primarily occurs within the colon^26,27^. Importantly, the rate-limiting step in bacterial BA metabolism is deconjugation of amino acids from the sterane core of BAs. This is carried out by a specific bacterial enzyme called *bile salt hydrolase (bsh)* that is distributed among a diverse array of bacterial taxa^28^. Dysregulation of BA metabolism has been linked to numerous inflammatory and metabolic diseases in humans^16,29,30^. Recently, ‘common variable immunodeficiency’ (CVID), the most commonly observed and clinically relevant form of antibody-deficiency in humans, has been linked to defects in lipid metabolism in the small intestine and BA malabsorption^11,12,31^, providing evidence to support that defects in mucosal antibody responses may perturb BA homeostasis in antibody-deficient humans.

Given that mucosal antibody responses shape microbiota composition, and microbiota composition in turn shapes BA biochemistry, we sought to address the hypothesis that dysbiosis caused by antibody-deficiency may promote disease by disrupting BA homeostasis in the gut. To address this hypothesis, we utilized a CD19^−/−^ mouse model of antibody-deficiency. The CD19 co-receptor is one of several key molecules whose collective actions facilitate proper B cell receptor signaling and ultimately B cell activation. Previous work using immunization models have shown that CD19^−/−^ mice have severe defects in their ability to mount antibody responses against systemically or orally administered T-cell-dependent and T-cell-independent antigens^32,33^. Using this model of antibody-deficiency, we have recently reported that CD19^−/−^ mice have lower IgA titers in feces, bind fewer commensal microbes with IgA, and develop gut dysbiosis^34^. These mice also develop a gluten-sensitive inflammatory enteropathy restricted to the small intestine (SI) that is associated with defects in lipid metabolism and BA absorption. Here, we demonstrate that defects in BA composition correlates with specific features of dysbiosis observed in these animals. Additionally, using several approaches, we show that adoptive transfer of functional B cells, direct manipulation of BA availability in the gut, and ablation of a key enzyme necessary for bacterial BA bio-transformation are all able to modulate the severity of SI enteropathy in CD19^−/−^ mice.

## Results

### Dysbiosis is associated with abnormal BA composition in the gut

**Fig. 1:**
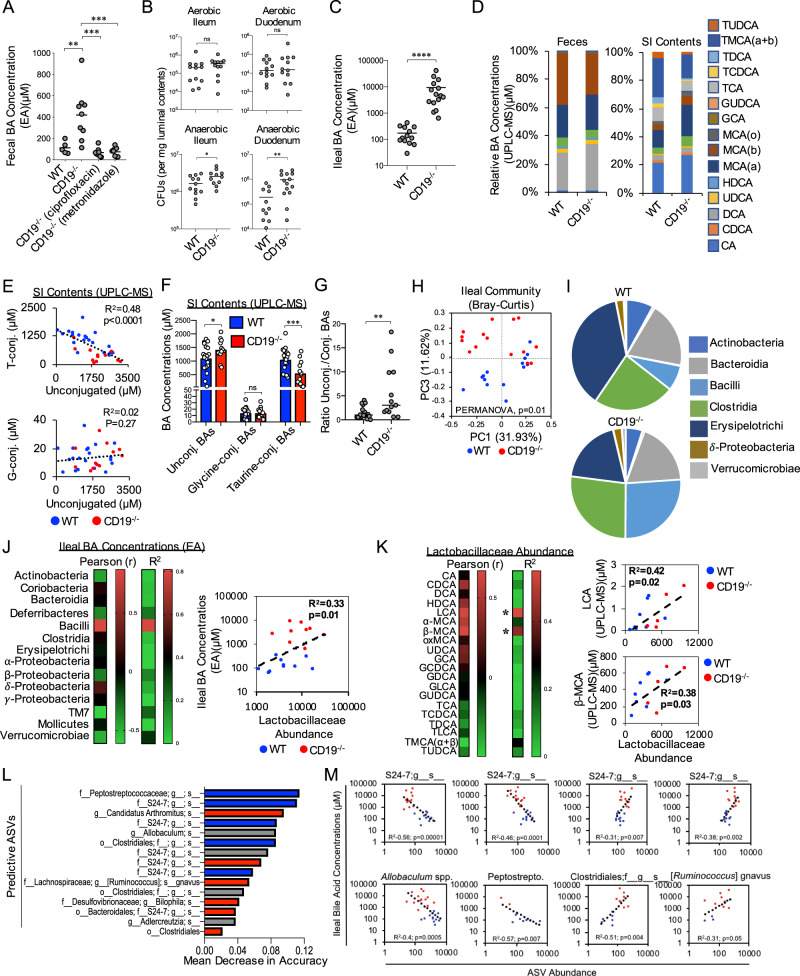
B cell deficiency is associated with altered BA composition in the gut. **A** Fecal BA concentrations of respective cohorts are shown. Tukey’s multiple comparisons test; (WT vs. CD19^−/−^), **=*p* = 0.0031, (CD19^−/−^ vs. CD19^−/−^ciprofloxacin), ***=*p* = 0.0001, (CD19^−/−^ vs. CD19^−/−^metronidazole), ***=*p* = 0.0002. Data representative of two replicate experiments. **B** Aerobic and anaerobic CFU measurements from ileum and duodenum are shown. Two-tailed unpaired Student’s t-test; ns = non-significant = *p* = 0.16, *=*p* = 0.05, **=*p* = 0.003. Data representative of three replicate experiments. **C** Ileal BA concentrations are shown. Two-tailed Student’s t-test; ****=*p* < 0.0001. Data representative of three replicate experiments. **D** Stacked bar-chart illustrating general shifts in BA abundance as measured by UPLC-MS. Source data are provided as a Source Data file. Data representative of three replicate experiments. **E** Correlation analysis of relationship between conjugated and unconjugated BA concentrations in the ileum of WT and CD19^−/−^ mice. (upper plot) Two-tailed one-way ANOVA, Fstat_(1,31)_ = 19.93, *p* < 0.0001. (lower plot) Two-tailed one-way ANOVA, Fstat_(1,31)_ = 1.263, *p* = 0.27. **F** The relative abundance of unconjugated and conjugated BAs in WT and CD19^−/−^ mice is shown. Sidak’s multiple comparisons test; ns = >0.99, *=*p* = 0.04, ***=*p* = 0.0004. **G** Comparison of the ratio of unconjugated to conjugated BAs between genotypes is shown. Two-tailed Student’s t-test; **=*p* = 0.002. **H** A PcoA plot based on Bray–Curtis distance of ileal microbial community composition is shown. Results of multivariate PERMANOVA hypothesis testing is shown (*p* = 0.01). **I** Pie-charts illustrating differential abundance of major bacterial Classes between WT (*n* = 14) and CD19^−/−^ (*n* = 15) mice are shown. Source data are provided as a Source Data file. **J** A heatmap summarizing descriptive statistics (Pearson r-coefficient and r-squared) of relationship between bacterial group abundance shown in (**E**) with associated ileal BA concentrations from the same mice (left panel). The correlation between Lactobacillaceae abundance and ileal BA concentrations are also shown (right panel). For right panel: two-tailed one-way ANOVA, *F*_(1,16)_ = 8.041, *p* = 0.01. **K** A heatmap summarizing descriptive statistics (Pearson r-coefficient and r-squared) of relationship between Lactobacillaceae abundance and the abundance of specific BAs as measured by UPLS-MS is shown. Right panel: LCA correlation-two-tailed one-way ANOVA, *F*(1,11) = 7.842, *p* = 0.02, β-MCA correlation-two-tailed one-way ANOVA, *F*(1,11) = 6.599, *p* = 0.03. **L** Bar-charts representing the top 15 bacterial groups influencing mean accuracy of Random Forest classification of samples by genotype (Blue bars: enriched in WT; Red bars: enriched in CD19^−/−^ mice). **M** Results of correlation analysis of ASV read abundance of differentially enriched bacterial groups in (**L**) demonstrating a significant association with ileal BA concentrations. Results of two-tailed one-way ANOVAs are shown in respective plots (each comparison stands alone). **J**, **K** Descriptive statistics were generated without adjustment for multiple comparisons.

Recently, our group demonstrated that outgrowth of obligate and/or facultative anaerobic bacteria (hereafter referred generally as “anaerobic” bacteria) in the feces of CD19^−/−^ mice was associated with elevated fecal BA concentrations^34^. To determine if bacterial outgrowth may be contributing to the observed alterations to fecal BA concentrations in CD19^−/−^ mice, we treated CD19^−/−^ mice for one week with a broad-spectrum antibiotic (ciprofloxacin) or an antibiotic that specifically restricts anaerobic bacterial growth (metronidazole) and then measured resulting fecal BA concentrations using an enzymatic assay (EA). We found that both antibiotic treatments significantly reduce total fecal BA concentrations in CD19^−/−^ mice to levels commensurate to concentrations observed in the feces of WT animals (Fig. 1A). Extending upon previous observations reported in the feces of CD19^−/−^ mice^34^, we now report that outgrowth of anaerobic bacteria (Fig. 1B) and elevated luminal BA concentrations (Fig. 1C) also extends into the SI of these animals. Furthermore, the previously described SI enteropathy in CD19^−/−^ mice appears to be restricted to the ileum (Supplementary Fig. S1), which is the major portal of BA re-absorption from the gut^35^. To more fully describe defects in BA homeostasis in CD19^−/−^ mice, we compared BA composition in the feces and SI of WT and CD19^−/−^ mice using ultra performance liquid chromatography mass spectrometry (UPLC-MS). Analysis of feces reveals no statistically significant differences in BA composition between WT and CD19^−/−^ mice (Fig. 1D and Supplementary Fig. S2, and Supplementary Data 1). However, analysis of SI luminal contents revealed significant shifts in the BA composition in CD19^−/−^ mice (Fig. 1D and Supplementary Data 1). Specifically, we observe a defect in the balance between unconjugated and taurine-conjugated BAs (but not glycine-conjugated BAs) in the SI of CD19^−/−^ mice (Fig. 1E). CD19^−/−^ mice were observed to have significant reductions in the relative abundance of taurine-conjugated BAs and consequent increases in unconjugated BAs (Fig. 1F and Supplementary Fig. S3). Additionally, we find that the ratios of unconjugated to conjugated BAs is increased in CD19^−/−^ mice suggesting that there may be elevated BA deconjugation by SI-resident bacteria in CD19^−/−^ mice (Fig. 1G). To more fully describe the dysbiosis observed in CD19^−/−^ mice, we performed 16S rRNA gene sequencing on ileal microbial communities. As we have previously reported, our new analysis again confirms that CD19^−/−^ mice develop a significantly altered ileal microbiota composition (Fig. 1H) (β-diversity, PERMANOVA, pseudo-Fstat_2,29_ = 2.91, *p* = 0.02), which was dominated by outgrowth of a number of anaerobic groups. At a broad level of taxonomic organization, CD19^−/−^ mice were observed to have increased abundance of members of the Class Bacilli (Fig. 1I and Supplementary Fig. S4A, B), and specifically members of Lactobacillaceae Family (Supplementary Fig. S4C). Despite this observation, however, no specific amplicon sequence variant (ASV; a.k.a “species”) within the Lactobacillaceae were significantly enriched in CD19^−/−^ mice (Supplementary Data 2), which implies a general inability to control the outgrowth of Lactobacillaceae family members. Overall, 58 specific ASVs were found to be differentially abundant in CD19^−/−^ mice compared to WT controls as estimated using discrete false discovery rate analysis (Supplementary Data 3), with the outgrowth of a number of ASVs within the Class Clostridia being a dominant feature (Supplementary Fig. S4D). Other notable anaerobes outgrown in CD19^−/−^ mice included segmented filamentous bacteria (SFB) and an unidentified species of bacteria within the genus *Bilophila* (Supplementary Fig. S4E). To determine if there is an association between microbiota composition and BA concentrations in the ileum of mice, we first performed correlation analysis between paired 16S and BA datasets. With respect to BA concentrations estimated using an enzymatic assay, Bacilli abundance was the only microbial feature we observed to be significantly positively correlated with ileal BA concentrations, and this effect was driven specifically by shifts in the relative abundance of Lactobacillaceae between genotypes (Fig. 1J). In general, Lactobacillaceae abundance was also found to be positively correlated with the abundance of unconjugated BAs and negatively correlated with the abundance of conjugated BAs (Fig. 1K). More specifically, significant positive correlations were observed between ileal Lactobacillaceae abundance and the concentrations of the unconjugated BAs lithocholic acid and β-muricholic acid (β-MCA) (Fig. 1K); two BAs that were observed to be significantly enriched in the SI contents of CD19^−/−^ mice (Supplementary Fig. S3).

A second approach that we applied to determine if there is an association between microbiota composition and BA concentrations in our animals was to identify which ASVs were most discriminatory between WT and CD19^−/−^ mice using two different approaches; Lefse and Random Forest Analysis. From these results, we then determined whether discriminatory ASVs were significantly correlated with ileal BA concentrations. Linear discriminant analysis using the Lefse pipeline identified eight species whose abundance were discriminatory between WT and CD19^−/−^ mice (Supplementary Fig. S5A). Notably, the five species outgrown in CD19^−/−^ mice were anaerobes or facultative anaerobes including *Akkermansia muciniphila*, *Alistipes indistinctus*, two unidentified species within the *Anaerostipes* and *Odoribacter* genera, as well as an unidentified species within the Class Alphaproteobacteria (Supplementary Fig. S5B). While the outgrowth of these species co-varied with increased BA concentrations in the ileum of CD19^−/−^ mice, there was no statistically significant correlation between the abundance of any of these eight species with ileal BA concentrations (Supplementary Fig. S5C). In contrast, Random Forest analysis, a machine learning technique that we used to identify the minimal set of bacteria that maximally predict host genotype, revealed numerous associations between the abundance of discriminatory bacterial species and ileal BA concentrations. Figure 1L reports the results of Random Forest analysis and summarizes the top fifteen most discriminating bacterial taxa that collectively predict mouse genotype (model (i.e., predictive) accuracy 83%). Eight of these fifteen discriminatory species were significantly correlated with ileal BA concentrations in mice (Fig. 1M). Collectively, these data indicate that a number of features of dysbiosis observed in CD19^−/−^ mice are strongly associated with abnormalities in the ileal BA pool.

### Ileal/Liver transcriptomics does not reveal major alterations to the expression of genes associated with BA metabolism, transport, and signaling in CD19^−/−^ mice

**Fig. 2:**
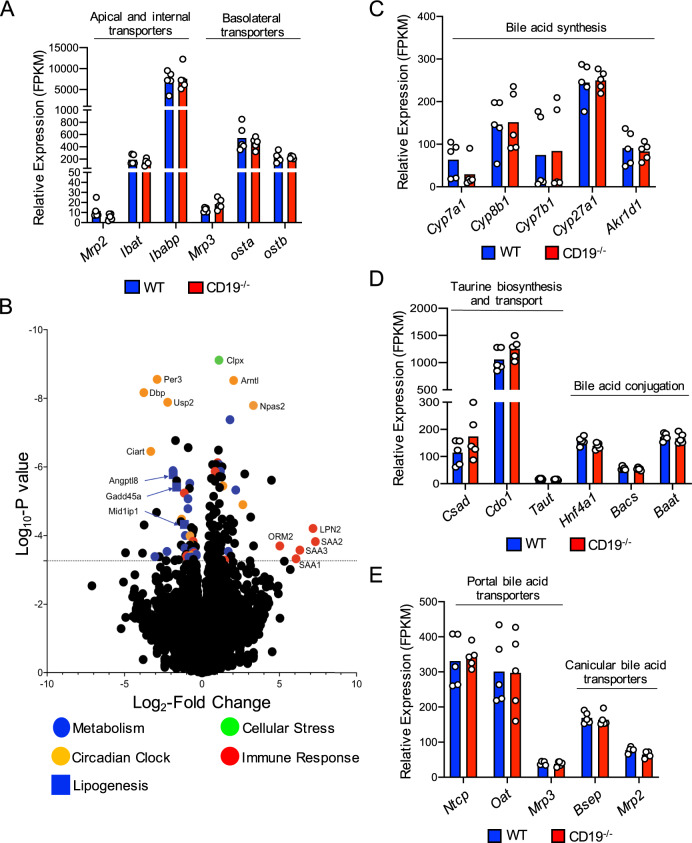
BA abnormalities are not due to host defects in Bile acid synthesis, conjugation, or reabsorption in CD19^−/−^ mice. **A** Gene expression of bile acid transporters in the distal ileum of mice. **B** Volcano plot of major differentially regulated genes in the liver of CD19^−/−^ mice compared to WT controls. The FDR-adjusted significance cutoff (FDR < 0.05) of quasi-likelihood hypothesis testing is illustrated by line along *Y*-axis. **C** Gene expression of liver enzymes involved in bile acid synthesis. **D** Gene expression of liver enzymes associated with taurine biosynthesis and bile acid conjugation. **E** Gene expression of liver bile acid transporters. **A**–**E** Data is representative of one ileal RNAseq and one liver RNAseq experiment each containing five biological replicates for each genotype. Significance determined by quasi-likelihood testing with an FDR significance cutoff of <0.05. None of the genes shown are differentially regulated based on FDR-adjusted p-values. Ileal and liver RNAseq data sets are derived from the same set of 5 female WT and 5 female CD19^−/−^ mice. Source data are provided as a Source Data file.

To determine if altered BA composition was due to defects in host BA metabolism or transport, we performed RNAseq to compare ileal and liver transcriptomes between WT and CD19^−/−^ mice. Within the ileum, we observe no differences in the expression of genes encoding transporters associated with apical or basolateral transport of BAs out of the ileum and into the portal vein (Fig. 2A and Supplementary Data 4). Within the liver, we find numerous genes are differentially regulated in CD19^−/−^ mice compared to WT mice (Fig. 2B and Supplementary Data 5). Consistent with the gene expression pattern previously reported in the ileum of CD19^−/−^ mice^34^, we observed that elevated expression of immune response genes was associated with decreased expression of a number of metabolic genes in the liver (Fig. 2B), consistent with a tradeoff between immune investment and metabolic function. Upregulated immune genes include genes that encode acute phase proteins (*SAA1*, *SAA2*, *SAA3*, *ORM2*, *LPN2*). Several circadian clock genes, which are important regulators of metabolic tone, are also differentially regulated in CD19^−/−^ mice. Additionally, three genes associated with lipid metabolism (*Angptl8*, *Gadd45a*, and *Mid1ip1*) are significantly downregulated in CD19^−/−^ mice, providing further support for our argument that CD19^−/−^ mice develop a lipid metabolic disorder. Interestingly, despite observed differences in BA composition in CD19^−/−^ mice, we do not observe any alterations in the expression of genes associated with the synthesis of BAs in the liver (Fig. 2C), the synthesis or conjugation of taurine to BAs in the liver (Fig. 2D), or the transport of BAs from the portal vein into the liver or the transport of BAs from the liver into the gall bladder (Fig. 2E and Supplementary Data 6). Finally, ileal and liver transcriptomic analyses failed to reveal any differences between WT and CD19^−/−^ mice in the expression of several BA receptors or key downstream signaling molecules including the major BA sensor *FXR* and the downstream signaling molecules *SHP* and *FGF15* (Supplementary Fig. S6A, B and Supplementary Data 4 and  6).

### SI enteropathy is associated with elevated IEC apoptosis

**Fig. 3:**
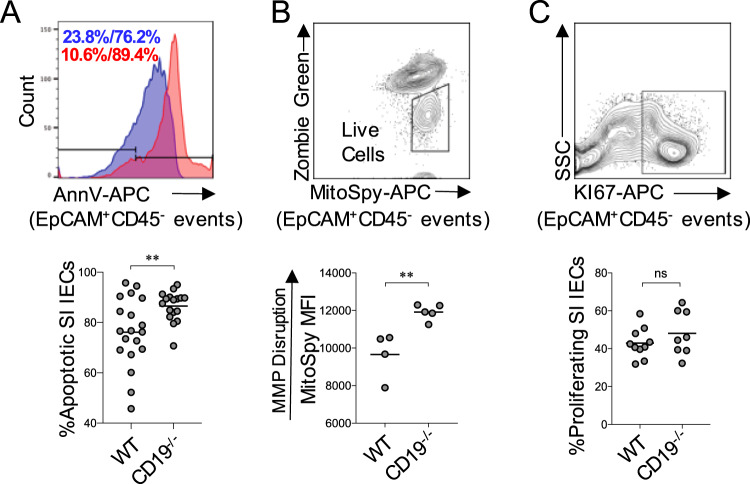
SI enteropathy is associated with increased IEC apoptosis in CD19^−/−^ mice. **A** Representative flow cytometry plot demonstrating gating on apoptotic IECs (EpCAM^+^CD45^−^PI^−^AnnV^+^) and the percentage of apoptotic IECs in WT and CD19^−/−^ mice are shown. Two-tailed unpaired Student’s t-test; **=*p* = 0.007. Data representative of three replicate experiments. **B** Representative flow cytometry plot demonstrating gating on live IECs (EpCAM^+^CD45^−^ZombieGreen^−^) and MitoSpy MFI values in live IECs in WT and CD19^−/−^ mice are shown. Two-tailed unpaired Student’s t-test; **=*p* = 0.006. Data representative of two replicate experiments. **C** Representative flow cytometry plot demonstrating gating on proliferating IECs (EpCAM^+^CD45^−^Ki67^+^) and the percentage of proliferating IECs in WT and CD19^−/−^ mice are shown. Two-tailed unpaired Student’s t-test; ns=non-significant=*p* = 0.28. Data representative of three replicate experiments.

CD19^−/−^ mice develop a chronic SI enteropathy that is histologically characterized by leukocytosis, inflammation, crypt hyperplasia, and villous blunting. BA biochemistry in the gut has a major influence on IEC turnover^23^, and the balance between IEC apoptosis and proliferation can influence all of these histological parameters. Therefore, we wanted to determine if rates of IEC proliferation or apoptosis were altered in CD19^−/−^ mice. To do this, we performed flow cytometry to compare the abundance of ileal IECs (EpCAM^+^CD45^−^) undergoing apoptosis (EpCAM^+^CD45^−^PI^−^AnnV^+^) (Supplementary Fig. S7) between WT and CD19^−/−^ mice. Results from our experiments revealed significantly enhanced apoptosis of IECs in CD19^−/−^ mice (Fig. 3A). Decreased mitochondrial health is a hallmark feature of apoptotic cells or cells undergoing cellular stress. To measure mitochondrial stress, we measured cellular uptake of a fluorescent dye (MitoSpy-NIL DilC(5)) whose intracellular concentration is positively correlated with disruption to mitochondrial membrane potential (MPP). Using this assay, we observed significantly elevated uptake of the fluorescent dye in live IECs (EpCAM^+^CD45^−^ZombieGreen^−^) from CD19^−/−^ mice (Fig. 3B). Finally, analysis of proliferation in IECs (EpCAM^+^CD45^−^Ki67^+^) revealed no differences between WT or CD19^−/−^ mice (Fig. 3C). Taken together, these data indicate that IEC health and turnover are perturbed in CD19^−/−^ mice.

### Manipulation of BA availability influences the severity of SI enteropathy

**Fig. 4:**
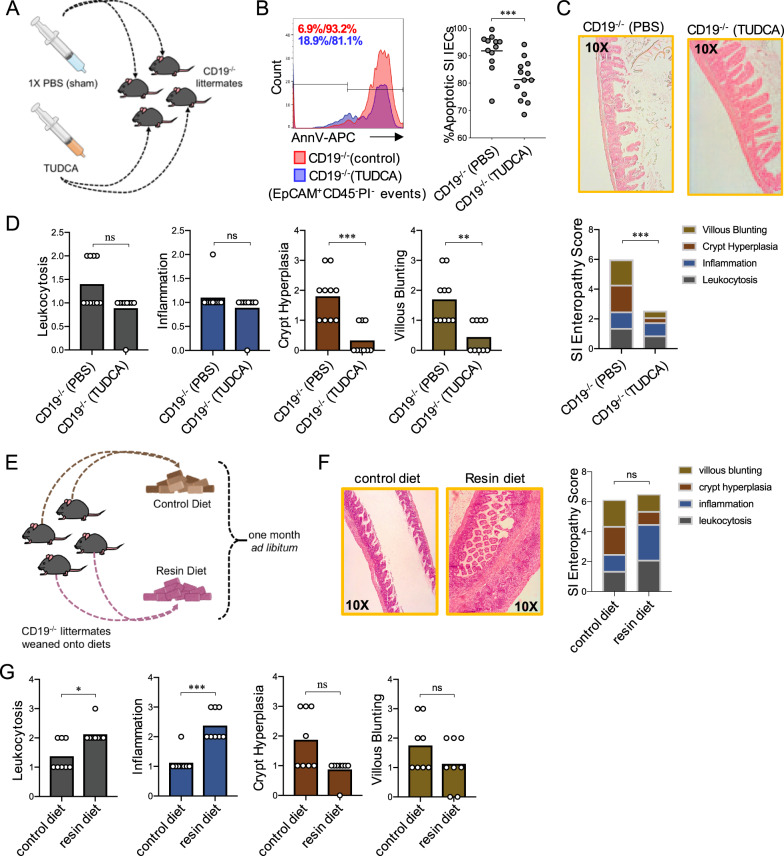
Manipulation of BA availability alters severity of SI enteropathy. **A** Schematic overview of oral TUDCA supplementation experiment. Syringe illustrations in (**A**) are image elements derived from BioRender.com. **B** Representative flow cytometry plots and comparison of the percentage of apoptotic IECs in vehicle (PBS) (*n* = 12) or TUDCA-treated (*n* = 13) CD19^−/−^ mice are shown. Two-tailed unpaired Mann–Whitney U test; ***=*p* = 0.0009. **C** SI enteropathy scoring is shown for vehicle or TUDCA-treated CD19^−/−^ mice. Two-tailed unpaired Mann–Whitney U test; ***=*p* = 0.0008. **D** Comparison of individual features of SI enteropathy between vehicle or TUDCA-treated CD19^−/−^ mice are shown. Two-tailed unpaired Mann–Whitney U test; (leukocytosis), n = non-significant =*p* = 0.0542, (inflammation), ns = non-significant =*p* = 0.53, **=*p* = 0.003, ***=*p* = 0.0008. **E** Schematic overview of cholestyramine diet exposure experiment. Mouse chow illustrations in (**E**) are image elements derived from BioRender.com. **F** Results of SI enteropathy scoring are shown for CD19^−/−^ mice exposed to control versus resin diet. Two-tailed unpaired Mann–Whitney U test; ns = non-significant =*p* = 0.66. **G** Comparison of individual features of SI enteropathy between CD19^−/−^ mice exposed to control versus resin diet are shown. Two-tailed unpaired Mann–Whitney U test; (crypt hyperplasia), ns=non-significant=*p* = 0.0513, (villous blunting), ns = non-significant =*p* = 0.27, *=*p* = 0.0.02, ***=*p* < 0.0009. **A**–**D** Data representative of three replicate experiments. **E**–**G** Data representative of two replicate experiments.

Deconjugation of the polar amino acid from the sterane core of BAs enhances their hydrophobicity and sub-micromolar increases in the abundance of hydrophobic BAs can be cytotoxic and induce apoptosis in IECs^36^. Given the observed defects in BA composition in the SI, we reasoned that SI enteropathy may be influenced by decreased availability of conjugated BAs. To test this, we performed two experiments where we directly manipulated the availability of luminal BAs. First, we performed an experiment where we enhanced the availability of conjugated BAs through oral supplementation. Here, CD19^−/−^ mice were treated with the hydrophilic taurine-conjugated BA species tauro-ursodeoxycholic acid (TUDCA) (Fig. 4A). We chose TUDCA for the following reasons. First, TUDCA is one of the most significantly reduced taurine-conjugated BA species observed in SI contents of CD19^−/−^ mice (Supplementary Fig. S3). Second, previous studies have shown that oral TUDCA supplementation can significantly reduce the severity of chemically-induced colitis in mice through it’s anti-apoptotic effect on IECs^37–39^. We administered TUDCA (500 mg/kg/day) to CD19^−/−^ mice via oral gavage daily for one week and then measured IEC apoptosis. We observed that seven days of oral TUDCA supplementation significantly reduced IEC apoptosis in our CD19^−/−^ animals (Fig. 4B). Moreover, in an independent experiment, we also observed that daily oral TUDCA supplementation for one week significantly reduces the severity of SI enteropathy in CD19^−/−^ mice (Fig. 4C and Supplementary Fig. S8), with the most pronounced effect being alleviation of crypt hyperplasia and villous blunting in TUDCA-treated CD19^−/−^ mice (Fig. 4D). To determine if reduced BA availability exacerbated disease, we exposed CD19^−/−^ mice to a diet containing 2% cholestyramine resin or a nutritionally-matched control diet (Fig. 4E). Cholestyramine resin is a BA chelator that causes BA malabsorption in mice^40^. One month post-exposure, animals were sacrificed and the degree of SI enteropathy was assessed. While cumulative SI enteropathy scores were not different between control and cholestyramine-fed mice (Fig. 4F), we found that the degree of leukocytosis and inflammation were significantly increased in resin-fed mice (Fig. 4G). Thus, exposure to cholestyramine resin diet, which exacerbates BA malabsorption, enhances inflammatory responses in CD19^−/−^ mice.

### Adoptive transfer of functional B cells alleviates SI enteropathy

**Fig. 5:**
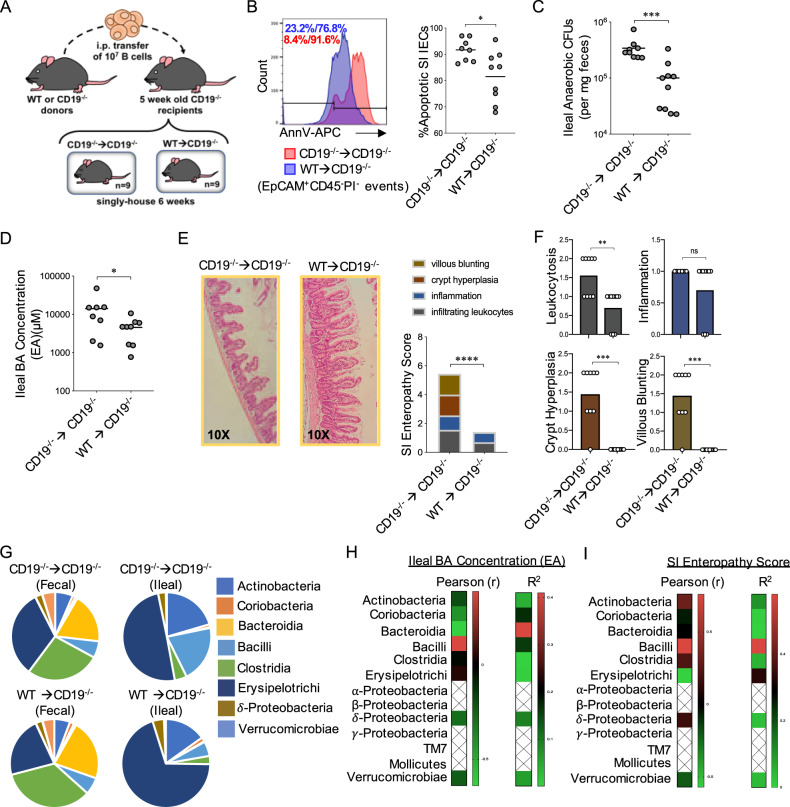
Adoptive transfer of WT B cells rescues CD19^−/−^ mice from SI enteropathy. **A** Schematic of adoptive transfer experiment. **B** Representative histogram plot demonstrating gating on apoptotic IECs and results of flow experiments are shown. Two-tailed unpaired Student’s t-test with Welch’s correction for uneven variance; *=*p* = 0.01. **C** Anaerobic CFU titers from ileal contents are shown. Two-tailed unpaired Mann–Whitney U test; ***=*p* = 0.0006. **D** Total ileal BA concentrations are shown. Two-tailed unpaired Student’s t-test; *=*p* = 0.05. **E** SI enteropathy scoring is shown for CD19^−/−^ mice receiving B cells from WT or CD19^−/−^ mice. Two-tailed unpaired Mann–Whitney U test; ****=*p* < 0.0001. **F** Comparison of individual features of SI enteropathy between CD19^−/−^ mice receiving B cells from WT or CD19^−/−^ mice. Two-tailed unpaired Mann–Whitney U test; ns = non-significant =*p* = 0.21, **=*p* = 0.004, ***=*p* = 0.0001 (crypt hyperplasia), ***=*p* = 0.0001 (villous blunting). **G** Pie-charts illustrating differential abundance of major bacterial Classes are shown. Source data are provided as a Source Data file. **H** Heatmaps summarizing descriptive statistics (Pearson r-coefficient and r-squared) of relationship between ileal bacterial group abundance and ileal BA concentrations are shown. **I** Heatmaps summarizing descriptive statistics (Pearson r-coefficient and r-squared) of relationship between ileal bacterial group abundance and SI Enteropathy severity are shown. **B**–**I** Data representative of two replicate experiments. **H**, **I** Hatched values indicate bacterial groups that were not found present in more than 75% of samples, and were thus excluded from analysis.

To explicitly determine if transfer of functional B cells could rescue observed phenotypic defects observed in CD19^−/−^ mice, we performed an experiment where we adoptively transferred equivalent numbers of splenic B cells from CD19^−/−^ or WT mice into CD19^−/−^ mice (Fig. 5A and Supplementary Fig. S9A). Six weeks post adoptive transfer of B cells, we observed that transfer of WT B cells reduced the abundance of apoptotic IECs (Fig. 5B), reduced the abundance of anaerobic bacteria in ileal contents (Fig. 5C) and reduced BA concentrations in the ileal contents of CD19^−/−^ recipients (Fig. 5D). WT B cell transfer also alleviated SI enteropathy (Fig. 5E) (Supplementary Fig. S9B). Leukocytosis, crypt hyperplasia, and villous blunting were all significantly reduced in CD19^−/−^ mice receiving WT B cells (Fig. 5F). To determine if the adoptive transfer of WT B cells into CD19^−/−^ mice alters microbiota composition, we performed 16S analysis on fecal and ileal communities of CD19^−/−^ mice that received WT or CD19^−/−^ B cells. Overall, fecal and ileal community composition (β-diversity) did not diverge between cohorts of CD19^−/−^ mice though a marginally-significant shift in composition could be observed between ileal communities (Supplementary Fig. S10A). Additionally, within the ileal (but not fecal) community, we observed a reduction in the abundance of bacteria within the Class Bacilli in CD19^−/−^ mice receiving WT B cells (Fig. 5G). Bacilli abundance was again the only microbial feature we observed to be positively associated (Pearson r) with ileal BA concentrations, though this relationship was not statistically significant (Fig. 5H). Bacilli abundance was also found to be significantly positively correlated with SI enteropathy scores in mice (Fig. 5I). The shift in ileal Bacilli abundance in our mice was driven by reductions in the abundance of members of the Lactobacillaceae family (Supplementary Fig. S10B and Supplementary Data 2), and the abundance of Lactobacillaceae bacteria was also found to be positively correlated with ileal BA concentrations (Supplementary Fig. S10C) and SI enteropathy scores (Supplementary Fig. S10D).

### Bacterial *bsh* activity is perturbed in the SI of CD19^−/−^ mice

**Fig. 6:**
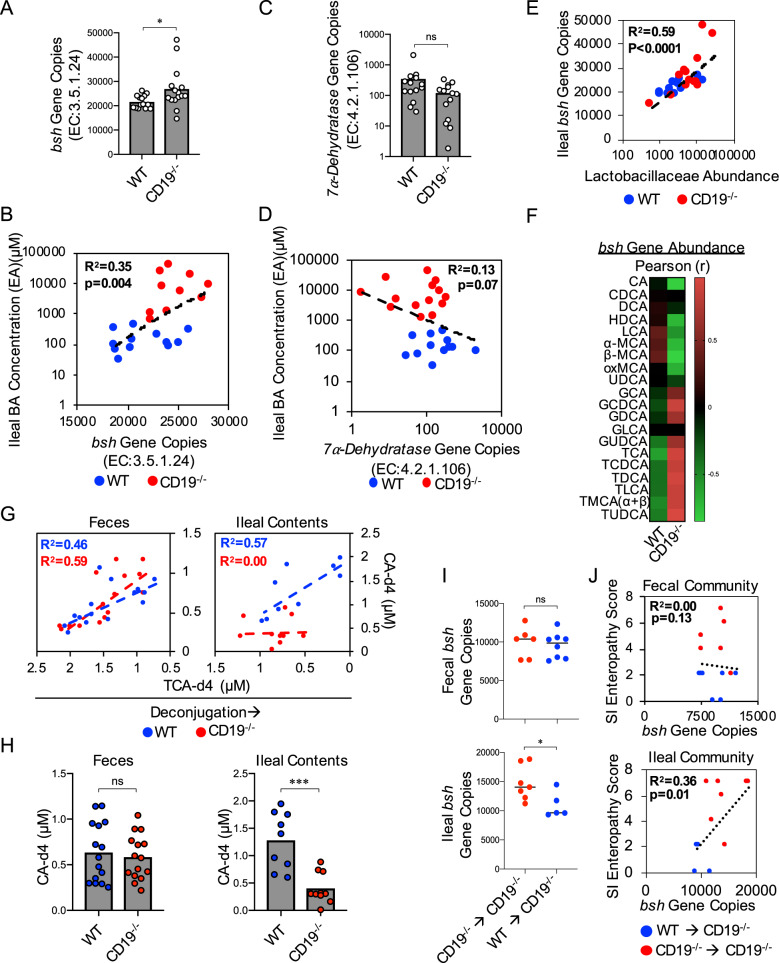
Ileal *bsh* activity is perturbed in CD19^−/−^ mice. **A** Total *bsh* (EC:3.5.1.24) gene abundance in WT and CD19^−/−^ mice is shown. Two-tailed unpaired Student’s t-test; *=*p* = 0.04. **B** Correlation between *bsh* gene abundance and ileal BA concentrations is shown. Two-tailed one-way ANOVA, Fstat(1,20) = 10.66, *p* = 0.004. **C** Total *7α-dehydratase* (EC:4.2.1.106) gene copies in WT and CD19^−/−^ mice are shown. Two-tailed unpaired Student’s t-test; ns = *p* = 0.0651. **D** Correlation between *7α-dehydratase* gene abundance and ileal BA concentrations is shown. Two-tailed one-way ANOVA, Fstat_(1,20)_ = 0.13, *p* = ns. **E** Correlation between Lactobacillaceae abundance and *bsh* gene abundance is shown. Two-tailed one-way ANOVA, Fstat_(1,23)_ = 33.48, *p* < 0.0001. **F** Heatmaps summarizing descriptive statistics (Pearson r-coefficient and r-squared) of relationship between ileal bacterial *bsh* gene abundance and specific BA species are shown. Data representative of two replicate experiments. **G** Correlation between TCA-d4 and CA-d4 abundance (μM) in fecal protein extracts (left panel) and ileal content protein extracts (right panel) from WT and CD19^−/−^ mice. Two-tailed one-way ANOVA (WT fecal protein extracts), Fstat(1,13) = 18.71_,_ *p* = 0.0008. Two-tailed one-way ANOVA (CD19^−/−^ fecal protein extracts), Fstat_(1,13)_ = 11.04_,_ *p* = 0.006. Two-tailed one-way ANOVA (WT ileal protein extracts), Fstat_(1,7)_ = 9.186_,_ *p* = 0.02. Two-tailed one-way ANOVA (CD19^−/−^ ileal protein extracts), Fstat(1,8) = 0.0006_,_ *p* = 0.98. **H** Total abundance of deconjugated CA-d4 in fecal and ileal content protein extracts from WT and CD19^−/−^ mice. Two-tailed unpaired Student’s t-test; ns = non-significant =*p* = 0.63, ***=*p* = 0.0002. **I** Total *bsh* gene copies in feces and ileal contents of CD19^−/−^ mice administered B cells from CD19^−/−^ or WT mice. Two-tailed unpaired Student’s t-test; ns = non-significant =*p* = 0.68, *=*p* = 0.04. **J** Correlation between SI enteropathy and *bsh* gene copies in feces or ileal contents of CD19^−/−^ mice administered B cells from CD19^−/−^ or WT mice. Two-tailed one-way ANOVA (feces), Fstat_(1,13)_ = 0.13_,_ *p* = ns. Two-tailed one-way ANOVA (ileal contents), Fstat_(1,10)_ = 7.61_,_ *p* = 0.01. **A**–**E** Data representative of three replicate experiments. **G**, **H** Data representative of two replicate experiments. **I**, **J** Data representative of two replicate experiments.

Bacteria encode several genes essential to BA metabolism in the gut, including the rate-limiting enzyme *bile salt hydrolase* (*bsh)* that is responsible for BA deconjugation^27,28^. Given the enhanced abundance of deconjugated BAs in the SI contents of CD19^−/−^ mice, we next compared the relative abundance of two key genes associated with bacterial BA metabolism between WT and CD19^−/−^ mice using PICRUSt2 predictive metagenomic analysis of our 16S datasets. CD19^−/−^ mice were observed to be enriched in ileal *bsh* gene abundance (Fig. 6A) and *bsh* gene abundance was also found to be positively correlated with ileal BA concentrations (ANOVA, F-stat_1,20_ = 10.66, *p* = 0.004) (Fig. 6B). However, the abundance of genes encoding the enzyme *7α-dehydratase* were similar between WT and CD19^−/−^ mice (Fig. 6C) and were not significantly correlated with ileal BA concentrations (ANOVA, Fstat_1,24_ = 3.58, *p* = 0.07) (Fig. 6D). A significant positive correlation was also observed between *bsh* gene abundance and Lactobacillaceae abundance in our animals (ANOVA, Fstat_1,23_ = 37.06, *p* = <0.0001) (Fig. 6E), implicating Lactobacillaceae family members as major contributors to the *bsh* gene pool. Interestingly, we found a significant difference in the relationship between *bsh* gene abundance and BA composition between WT and CD19^−/−^ mice. Specifically, we observed an inverse correlation between *bsh* gene abundance and conjugated BAs in WT mice, whereas in CD19^−/−^ mice we find a positive correlation between *bsh* gene abundance and the concentrations of conjugated BAs (Fig. 6F). Collectively, these data implied that ileal *bsh* activity may be abnormal in CD19^−/−^ mice. To test this explicitly, we next measured *bsh* activity by spiking standard concentrations of fecal or ileal protein extracts with a model taurine-conjugated BA (TCA-d4) and measured the resulting relative abundance of it’s deconjugated form (CA-d4). There was a significant inverse correlation observed between TCA-d4 and CA-d4 abundance in WT mice in both fecal and ileal protein extracts, whereas *bsh* activity is perturbed in ileal (but not fecal) protein extracts in CD19^−/−^ mice (Fig. 6G). Fecal CA-d4 abundance was consistent between genotypes, whereas ileal CA-d4 abundance was significantly reduced in CD19^−/−^ mice (Fig. 6H). Because of the observed difference in ileal BA concentrations in CD19^−/−^ mice administered WT or CD19^−/−^ B cells, we also compared ileal *bsh* gene abundance in these cohorts. Fecal *bsh* gene abundance was consistent between cohorts while ileal *bsh* gene abundance was observed to be lower in CD19^−/−^ mice receiving WT B cells (Fig. 6I). Finally, we also observed a significant correlation between ileal (but not fecal) *bsh* gene abundance and the severity of SI enteropathy that develops in mice (Fig. 6J).

### Ablation of bacterial *bsh* activity alleviates SI enteropathy

**Fig. 7:**
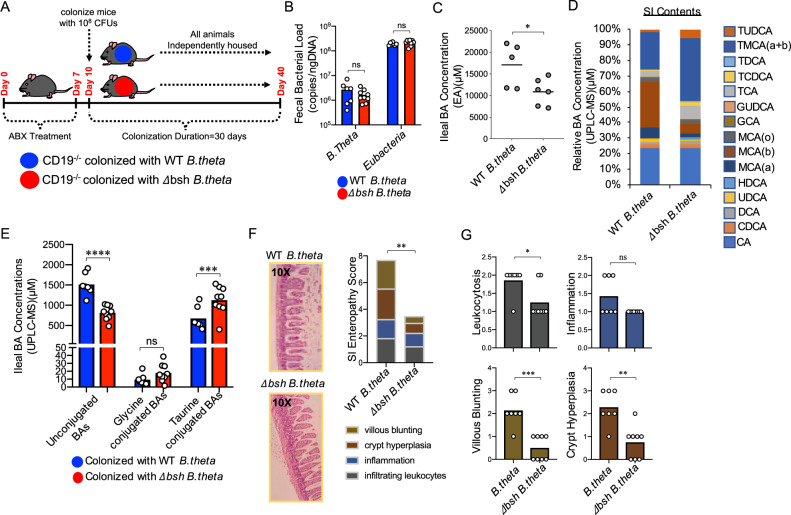
Microbial *bsh* activity drives BA defects and contributes to SI enteropathy in CD19^−/−^ mice. **A** Schematic overview of *B.theta* and *Δbsh B.theta* colonization experiments. **B** qPCR estimates of the relative abundance of *B.theta*, *Δbsh B.theta*, and total *Bacteroides* are shown. Sidak’s multiple comparisons test; *B.theta*, ns = *p* = 0.9972, Eubacteria, ns = *p* = 0.1483. **C** Ileal BA concentrations in *B.theta*-colonized *and* *Δbsh B.theta*-colonized CD19^−/−^ mice are shown. Two**-**tailed unpaired Student’s t-test; *=*p* = 0.03. **D** Stacked bar-chart illustrating general shifts in BA abundance as measured by UPLC-MS. Source data are provided as a Source Data file. **E** The relative abundance of unconjugated and conjugated BAs in *B.theta* and *Δbsh B.theta*-colonized CD19^−/−^ mice are shown. Sidak’s multiple comparisons test; ns = *p* = 0.9998, ***=*p* = 0.0007, ****=*p* < 0.0001. **F** SI enteropathy scoring is shown for CD19^−/−^ mice colonized with WT *B.theta* (*n* = 7) or *Δbsh B.theta* (*n* = 8). Two-tailed unpaired Mann–Whitney U test; **=*p* = 0.002. **G** Comparison of individual features of SI enteropathy between CD19^−/−^ mice colonized with WT *B.theta* or *Δbsh B.theta*. Two-tailed unpaired Mann–Whitney U test; ns = non-significant =*p* = 0.08, *=*p* = 0.04, **=*p* = 0.004, ***=*p* = 0.0008. **B**–**G** Data representative of two replicate experiments.

Given the observed abnormalities in *bsh* activity in CD19^−/−^ mice, and positive correlation between *bsh* gene abundance and SI enteropathy in our B cell transfer model, we sought to directly address whether bacterial *bsh* activity influences the development of SI enteropathy. To do this, we treated cohorts of CD19^−/−^ mice with antibiotics (ciprofloxacin and metronidazole) to deplete the microbiota in the gut and then colonized animals with one of two isogenic strains of the model commensal bacterium *Bacteroides thetaiotamicron*; one possessing intact *bsh* metabolic capacity (WT *B.theta*) or one that does not (Δ*bsh B.theta*) (Fig. 7A)^41^. Oral gavage of 10^8^ CFUs of either *B.theta* strain results in persistent and equivalent colonization of the gut of CD19^−/−^ mice up to 30 days post-colonization (Fig. 7B). Colonization of CD19^−/−^ mice with the mutant Δ*bsh B.theta* strain resulted in an apparent reduction in total ileal BA concentration (Fig. 7C) as well as an anticipated shift in BA composition in SI contents of CD19^−/−^ mice (Fig. 7D). As expected, we observed significant reductions in deconjugated BAs and a commensurate increase in the abundance of taurine-conjugated BAs in CD19^−/−^ mice colonized with Δ*bsh B.theta* (Fig. 7E, Supplementary Fig. S11 and Supplementary Data 1). Importantly, this was associated with alleviation of SI enteropathy in CD19^−/−^ mice (Fig. 7F and Supplementary Fig. S12), with significant reductions in leukocytosis, crypt hyperplasia, and villous blunting (Fig. 7G).

## Discussion

The microbiome profoundly influences BA biochemistry in the gut^27^, and gut humoral immunity regulates the spatial organization and metabolic function of the microbiota. Therefore, it is possible that dysbiosis caused by humoral immune deficits could drive gastrointestinal disease by altering BA homeostasis. Here, using a recently described CD19^−/−^ mouse model of SI enteropathy, we have performed a series of experiments that sought to address this hypothesis. Results from our experiments indicate that microbial dysbiosis that develops in CD19^−/−^ mice is correlated with defects in BA composition in the SI. Additionally, we demonstrate that the severity of SI enteropathy is sensitive to BA composition and BA availability supporting that SI enteropathy is a symptom of abnormal BA homeostasis in CD19^−/−^ mice. Using an adoptive transfer model, we demonstrate that reconstitution of CD19^−/−^ mice with functional B cells can reduce bacterial overgrowth and BA concentrations in the SI and alleviate SI enteropathy. Finally, colonization experiments of CD19^−/−^ mice with isogenic WT and *bsh*-null *B.thetaiotamicron* strains, we were able to directly demonstrate that bacterial *bsh* activity plays an important role in the development of SI enteropathy in our CD19^−/−^ mouse model. Collectively, these results support a model whereby dysbiosis caused by antibody-deficiency results in enhanced bacterial *bsh* activity in the SI. This in turn serves as a predisposing factor to inflammatory disease of the SI by causing defects in SI BA composition.

The role adaptive immunity plays in the regulation of BA metabolism has not been studied. Our adoptive transfer model sought to address the hypothesis that reconstitution of CD19^−/−^ mice with functional B cells would rescue animals from SI enteropathy, which is what we observed. Rescue from disease was associated with reduced abundance of anaerobic bacteria in the SI, altered microbiota composition, reduced ileal BA concentrations, and decreased bacterial *bsh* gene abundance. While anticipated, whether or not protection from disease is dependent upon antibody secretion by B cells remains unknown and is an important area for future investigation. Indeed, B cells can also protect from gastrointestinal inflammation through mechanisms independent of antibody production. For example, SAMP1/Yit mice are a model of Crohn’s disease that develops an SI enteropathy localized to the ileum, and disease has previously been shown to be associated with reduced IL10 secretion by B cells in mesenteric lymph nodes^42^. Additionally, CD19^−/−^ mice have been shown to be deficient in IL10-secreting B cells (so-called regulatory B cells) and using an adoptive transfer model Yanaba et al. were able to demonstrate that transfer of IL10-secreting B cells into CD19^−/−^ mice were able to protect them from DSS-induced colitis^43^. In this model, animals were administered DSS 48 h after adoptive B cell transfer and disease induction lasted seven days. Thus, the transfer of IL10-secreting B cells can rapidly exert a protective effect during an acute model of chemically-induced colitis. Similar results have also been obtained using a T cell transfer model of colitis^44^. These studies imply that B-cell-intrinsic IL10 production may be playing a protective role in our model of SI enteropathy as well. Ongoing adoptive transfer experiments using our CD19^−/−^ model that directly manipulates antibody secretion or the secretion of specific isotypes, as well as IL10 secretion by B cells, are being conducted to address these alternative hypotheses. Additionally, we are employing complementary mouse models of B-cell and antibody-deficiency to test the generality of the link between deficits in humoral immunity and BA metabolism.

A major host modification to nascently produced BAs in the liver is conjugation to the amino acids taurine and glycine which decreases BA hydrophobicity thereby enhancing their absorbability^45,46^. Our data support that B-cell-deficiency disrupts bacterial ecology in the SI which leads to enhanced *bsh* activity in the ileum. This in turn alters BA biochemistry and drives the SI enteropathy observed in our CD19^−/−^ mice. Other studies have shown that bacterial *bsh* activity can have a significant effect on host physiology. For example, over-expression of *bsh* genes cloned from different strains of *Lactobacillus salivarius* in the *bsh*-deficient K-12 strain of *Escherichia coli* was shown to differentially influenced weight gain, serum lipid profiles, and the expression of multiple genes associated with lipid metabolism in the SI of WT C57BL/6 mice^47^. More recently, it was shown that loss of *bsh* activity in *B.thetaiotamicron* reduced weight gain in C57BL/6 mice and also altered the expression of numerous metabolic genes in the SI^41^. Here, we provide evidence that *bsh* activity may play a pathogenic role. Results from our experiments indicate that outgrowth of anaerobic bacteria is associated with increased abundance of *bsh* genes in the SI of CD19^−/−^ mice, that *bsh* gene abundance is correlated with the severity of SI enteropathy, and that transfer of functional B cells reduces *bsh* gene abundance in the SI. Most importantly, direct ablation of bacterial *bsh* activity was shown to protect CD19^−/−^ mice from disease. There is precedent for believing similar processes may be occurring in humans. For example, expansion of bacteria in the SI is a clinically relevant disorder in humans that is termed ‘small intestinal bacterial overgrowth’ (SIBO). Interestingly, SIBO has been estimated as occurring in up to 5–15% of otherwise healthy humans^48^, which if accurate would mean that SIBO is one of the most common forms of dysbiosis. SIBO is associated with diarrhea, abdominal bloating, and in severe cases malabsorption and inflammatory disease. CVID patients may be at an elevated risk of developing this disorder^1^. Interestingly, SIBO can be artificially induced through the administration of acid-reducing drugs (e.g., cimetidine and omeprazole), and previous studies have shown that this results in enhanced bacterial deconjugation of BAs in the SI that could be treated with antibiotics^49–51^. Collectively, our results suggest that enhanced *bsh* activity in the SI may be a driver of disease in the setting of B-cell-deficiency. Recently, the development of a selective pan-*bsh* inhibitor that has been shown to abolish *bsh* activity within complex microbial communities in vivo^52^ will allow us to address whether this is a therapeutically targetable pathway.

A major unanswered question that arises from our study is how does alteration to luminal BA composition drives SI enteropathy. Importantly, both conjugated and unconjugated BAs are synthesized and secreted by the host into the gut under normal physiological conditions. However, disruption to physiological concentrations of both conjugated and unconjugated BAs can have detrimental effects on host physiology, and bacterial deconjugation of BAs directly influences this balance. There are several plausible explanations for how bacterial-driven alterations to luminal BA composition could drive disease. First, enhanced bacterial deconjugation could produce a more cytotoxic composition of BAs in the gut lumen. Several studies have shown that deconjugation of BAs enhances BA hydrophobicity, which destabilizes cellular membranes and promotes IEC apoptosis^19,23,27,30,53,54^. This could degrade barrier integrity and result in chronic immune activation driven by leakage of bacterial products from the gut into underlying tissues. Second, bacterial deconjugation of BAs may also produce an intrinsically more pro-inflammatory composition of BAs. Indeed, there is accumulating evidence that BAs serve as signaling molecules important for the induction of immunological tolerance in the gut. For example, signaling through the BA sensing receptors FXR and TGR5 have been shown to tolerize gut macrophages to chronic LPS stimulation^25^. Thus, it is possible that BA malabsorption diminishes this tolerizing signal or that deconjugated BAs are less effective at stimulating a tolerogenic program in gut macrophages. More recently, a link between bacterial BA metabolism and the generation of tolerogenic T cell responses was demonstrated. Song et al. were able to show that BAs were critical dietary factors promoting the development of a Rorγ + subset of regulatory T cells (Rorγ + Tregs) in the colonic LP of mice^24^. These researchers were also able to demonstrate, using a *bsh*-null strain of *B.thetaiotamicron*, that bacterial BA deconjugation was important for Rorγ + Treg development. Therefore, it is also possible that aberrant *bsh* activity may disrupt T cell homeostasis in the SI of CD19^−/−^ mice. Resolving the contribution of these potential two modes of pathogenesis (i.e., direct cytotoxicity to IECs or instigating inflammatory responses) will require determining whether SI enteropathy is driven by enhanced concentrations of unconjugated BAs or decreased concentrations of conjugated BAs in the gut lumen. Another challenge lies in determining whether the disease is a consequence of enhanced BA concentrations in the gut lumen, or decreased concentrations of re-absorbed BAs in underlying host tissues. While addressing these difficult questions is beyond the scope of the current study, they are an ongoing focus of our lab.

Abnormal BA biochemistry in the gut may also favor the outgrowth of pathobionts that drive the disease observed in our model. For example, SFB is a known pathobiont and previous work has suggested that this species may possess a functional *bsh* gene which could enhance its survival within an environment high in BAs^55^. Additionally, taurine is rich in sulfur and members of the sulfur-reducing Desulfovibrionaceae, including members of the genus *Bilophila*, are known to be able to utilize free taurine as an energy source^56^. Therefore, perhaps liberation of free taurine through BA deconjugation permits outgrowth of sulfur metabolizing pathobionts in the SI that drive inflammatory responses. Finally, contrary to conventional thought, it has recently been described that in both mice and humans, specific bacteria are actually capable of synthesizing novel amino acid-BA conjugates (phenylalanine-, tyrosine-, and leucine-conjugations)^57^. More importantly, increased abundance of these bacteria-derived conjugated BA species was associated with inflammatory bowel disease in humans as well as high-fat diet exposure in mice. Previously, we have described that CD19^−/−^ develops a lipid malabsorption characterized by increased fecal fat concentrations^34^. Therefore, we cannot rule out that novel bacteria-derived conjugated BA species (which were not quantified in this study) may also contribute to SI enteropathy.

Disruption to hepatobiliary circulation of BAs and BA metabolism results in numerous metabolic syndromes and inflammatory diseases in humans^23,30,58,59^. Additionally, a role for dysbiosis in driving pathogenic shifts in BA composition in the human gut has been demonstrated. For example, a study in humans has recently demonstrated that dysbiosis that emerges within ulcerative colitis (UC) patients that have had ileal-loop re-sectioning of their gut results in inflammation (pouchitis) due to a reduced capacity of the UC-pouch microbiome to synthesize secondary BAs^60^. There is some evidence to support that antibody-deficiency in humans perturbs BA homeostasis and that this may be a contributing factor to gastrointestinal disease. For example, the most common clinical symptom of BA malabsorption is diarrhea, and diarrhea is commonly observed in antibody-deficient humans^61^. Importantly, a recent study has reported that approximately 40% of CVID patients presenting with non-infectious diarrhea had defects in their ability to absorb BAs^31^. Additionally, prior work has shown a link between defects in lipid metabolism in the SI with the development of an SI-specific inflammatory disease (termed CVID enteropathy) that develops in a subset of CVID patients^11,12^. While BA phenotypes were not assessed in these studies, observed defects in SI lipid metabolism suggest they are likely to be abnormal. The current treatment for CVID in humans is recurring intravenous IgG infusions. However, IgG infusions are generally not effective in the treatment of non-infectious gastrointestinal complications associated with CVID^62^. Thus, there is a need to identify alternative strategies for the treatment of non-infectious enteropathies in this patient cohort. Dietary manipulation of BA biochemistry in the gut may be a novel therapeutic option, and for this reason, we believe consideration of a potential link between BAs and gastrointestinal disease is warranted in future clinical studies.

There are several limitations to our study that we would like to acknowledge. First, while we show that increased deconjugation of taurine-conjugated BAs is associated with SI enteropathy in our CD19^−/−^ mouse model, it is important to acknowledge that glycine-, rather than taurine-conjugated BAs, are the dominant conjugated BA species found in the human gut. Therefore, extrapolation of the results of our experiments to human disease need to be cautioned until this phenomenon is addressed in humans. Second, our data suggest that members of the Lactobacillaceae may be playing a prominent role in the phenotypes we describe, though our 16S data did not allow us to identify a specific species within this group that was consistently divergent between treatments (host genotype or B cell transfer). Regardless, there is precedent for believing that members of the *Lactobacillus* genus may be particularly relevant to our model. For example, a previous study found that enrichment of *Lactobacillus johnsonni* resulted in altered BA profiles in the mouse gut with preferential deconjugation of Tauro-β-muricholic acid^63^. Additionally, specific strains of *L.reuteri*, *L.vaginalis*, and *L.salivarius* (three species we do observe in our experimental cohorts) have all been shown to possess *bsh* enzymes^64^. In addition to members of the Lactobacillaceae, it is important to note that *bsh* genes are conserved across evolutionarily divergent bacterial clades found within the gut^28^. Because of this, we would argue that a diverse array of taxonomically distinct species may be able to exert the same effect if their abundance cannot be properly regulated by the immune system. Third, the results of the *bsh* activity assay indicate that *bsh* activity is altered in a site-specific manner in CD19^−/−^ mice. However, the low abundance of deconjugated CA-d4 in CD19^−/−^ mice appears at first-blush to contradict the idea that CD19^−/−^ mice have enhanced BA deconjugation in their ileum. We believe this result could be explained by an effect of differential bacterial BA substrate-specificity between WT and CD19^−/−^ mice. For example, while *bsh* genes are widely distributed across most commensal bacterial clades^28^, it has long been known that different bacterial species possess *bsh* enzymes that preferentially utilize different BAs as substrates. Thus, it is possible that the bacterial species outgrown in the ileum of CD19^−/−^ mice may not preferentially utilize taurocholic acid (TCA) as a substrate while those in WT mice do. Another possible explanation for this discrepancy could have to do with enhanced activity of other bacterial BA-modifying enzymes that more readily modify and transform available cholic acid (CA-d4). For example, while we observe similar abundance of inferred *7α-dehydratase* genes in WT and CD19^−/−^ mice, it is possible that enzyme activity is different between genotypes and that CA-d4 is more rapidly metabolized into secondary BA metabolites in CD19^−/−^ mice. Moreover, PICRUSt is a predictive metagenomics approach that uses 16S rRNA gene copy data to infer the functional gene content of a microbial community. It does not directly quantify gene abundance or gene activity, so it is likely that other BA-modifying enzymes are playing a role that cannot be appreciated in the current study. Finally, all of the mice in our colony are reared on a standardized mouse chow that contains gluten, and we have previously shown that the SI enteropathy that develops in CD19^−/−^ mice is gluten-sensitive as administration of a gluten-free diet reduces disease severity. Thus, it is important to acknowledge that dietary gluten plays a currently unappreciated pathological role in our model. However, the gluten-sensitive nature of the SI enteropathy we have described in CD19^−/−^ mice is dependent on humoral immune deficits as we have previously shown that WT mice maintained on the same diet do not develop enteropathy^34^. Furthermore, despite being maintained on the same gluten-containing chow, we show here that reconstitution of CD19^−/−^ mice with WT B cells rescues animals from disease. Because exposure to a gluten-containing chow facilitates the study of SI enteropathy in our model by exacerbating the disease phenotype, all mice used in the current study were maintained on a gluten-containing chow, and experimental cholestyramine diets were also formulated to contain the same amount of gluten. Therefore, while we cannot rule out the possibility that the differences we observe between WT and CD19^−/−^ mice in the current study may be influenced by gluten sensitivity, gluten sensitivity cannot explain the differences in BA composition and disease susceptibility we demonstrate between experimental cohorts of CD19^−/−^ mice in response to manipulation of BA availability, the presence of functional B cells, and *bsh* function because all of these mice were maintained on the same gluten-containing diet.

Results from our experiments are significant because they demonstrate that B cells play a role in BA homeostasis in the gut, which has not been previously described. Furthermore, they identify a specific element (*bsh* activity) of a common form of dysbiosis (SIBO) that may contribute to the pathogenesis of SI enteropathy. The relevance of these results extend beyond the context of primary antibody-deficiencies. For example, the incidence of SIBO in patients with Crohn’s disease (another inflammatory SI enteropathy) has been estimated to be as high as 30%^65^. Importantly, a recent meta-analysis also reported a SIBO incidence rate of 20% in patients suffering from celiac disease (CeD)^66^; the classic gluten-sensitive SI enteropathy. Results from our previous work^34^ and the work shown here indicate that dietary gluten, B cells, and BAs all play a role in determining the severity of SI enteropathy. Our work suggests that antibody deficiency is a predisposing factor for gluten sensitivity and this is consistent with the evidence in humans. For example, it has been estimated that CeD patients are 10–15 times more likely to have an associated IgA deficiency^67^. Defective B cell responses in the SI could promote gluten-sensitive enteropathies by leading to a breakdown in immunological tolerance in the gut (e.g., through decreased IL10 secretion or reduced BA signaling in gut-resident immune cells), or by modifying the composition of the microbiota. To the latter point, recent evidence supports that bacterial transglutaminase, a widely used additive in the food industry but also an enzyme found in commensal bacteria in the gut, may enhance the immunogenicity of gluten antigens^68^. Perhaps SIBO also results in the expansion of bacteria with this metabolic capacity in the SI. While beyond the scope of this study, understanding the link between humoral immune deficits and gluten sensitivity is an important avenue of future research. If these various forms of inflammatory disease are associated with enhanced *bsh* activity in the SI then our data suggest that oral BA supplementation or *bsh* ablation may represent novel therapeutic treatments of these SI-specific diseases.

## Methods

### Mouse models, husbandry, and diet

A breeding colony of WT C57BL/6 and CD19^−/−^ mice have been maintained by the Kubinak Lab at the University of South Carolina for two years. WT C57BL/6 mice (Jax#000664) and CD19^−/−^ mice (C57BL/6 background) (Jax#006785) were originally purchased from Jackson laboratories and were crossed to generate CD19^+/−^ F_1_ heterozygotes, which were subsequently bred to rederive homozygote WT and CD19^−/−^ breeding lines. All animals used in the experiments described here are derived from this colony. Eight to sixteen week-old age-matched male and female mice were used in all experiments. Animals were reared and maintained under identical SPF conditions in a single environmentally-controlled room exclusively used to house this mouse colony. Animals are maintained under constant environmental conditions (70 °F, 50% relative humidity, 12:12 light:dark cycles) and are given *ad libitum* access to autoclaved drinking water and an irradiated soy-free mouse chow containing gluten (Envigo; diet#2920X). All animal use strictly adhered to federal regulations and guidelines set forth by the University of South Carolina Institutional Animal Care and Use Committee, and the studies were approved by the South Carolina Institutional Animal Care and Use Committee (Protocol#101580).

### TUDCA treatment

Tauro-ursodeoxycholic acid (TUDCA) was purchased from Sigma-Aldrich (cat#580549) and was suspended in sterile 1X PBS. For in vivo use, animals were administered TUDCA at a concentration of 500 mg/kg/day for five sequential days via oral gavage. Seven days post-TUDCA administration animals were sacrificed and tissues were collected for downstream analysis.

### Cholestyramine treatment

Two mouse chows were purchased from BioServ to conduct cholestyramine experiments. Each chow was formulated to contain a similar amount of gluten as found in the standard mouse chow. Our experimental chow was supplemented with 2% cholestyramine (Sigma, C4650). A nutritionally-matched chow that lacked cholestyramine was formulated and served as our control diet. Animals were maintained until weaning age on the standard mouse chow. At four weeks of age, littermate male and female CD19^−/−^ mice were randomly assigned to receive one of the two diets. Animals were given *ad libitum* access to their respective diet for one month and were singly-housed for the duration of diet exposure. One month post-exposure, animals were sacrificed and disease severity was scored by a blinded pathologist.

### IEC isolation

IECs were collected as follows. The proximal 15 cm of small intestine were collected from mice. Luminal contents were squeezed from the gut tube and Peyer’s patches were removed. The gut tube was then splayed open with scissors and the luminal face of the gut epithelium was gently scraped to remove mucus and remaining luminal contents. Tissues were then finely minced with scissors and tissue was placed in a 50 mL conical containing 15 mL of cell dissociation solution (HBSS without Ca^++^ with 5 mM EDTA and 1 mM DTT). Conical tubes containing tissues were laid on their side and tissues were incubated at 37 °C for 30 min with rotation (200RPM). Conical tubes containing dissociated tissues were then vortexed for 20 s and tissue was passed through a 100 μm cell strainer placed inside a 50 mL conical tube. Flow through (containing IECs) were spun at 2000RPM to pellet. Cell pellets were re-suspended in complete RPMI solution (supplemented with FBS, sodium pyruvate, non-essential amino acids, L-glutamine, penicillin-streptomycin, and β-ME) and spun at 1350RPM for five minutes to wash the cells. Two washes were performed. Cells were then surface stained and analyzed by flow cytometry.

### Flow cytometry

Flow cytometry was performed on a BD Aria II cell sorter or BD Accuri C6 cytometer. To enumerate IECs, 500,000 cells per animal were stained with the following antibodies (anti-mouse CD326 (EpCAM), anti-mouse CD45, Propidium Iodide (PI), Annexin V, and anti-mouse Ki67). All antibodies were used at a final concentration of 1:250. Cells were stained in 100 μL volumes in the dark for 20 min. Stained cells were then washed twice with 1× wash buffer. Cells were then resuspended in 2% PFA for fixation. For Ki67 staining, surface stained cells (EpCAM and CD45) were stained with anti-mouse Ki67 antibody at a concentration of 1:50. Cells were stained in the dark at room temperature for 30 min, then washed twice in 1X wash buffer, and finally suspended in 2% PFA for fixation. For MitoSpy staining of mitochondria, isolated cells were subsequently processed as follows. For MitoSpy staining, IECs were harvested from the distal 15 cm of the small intestine and stained for 20 min with Zombie Green viability dye (1:500) at 37 °C with 5% CO_2_. Cells were then washed with column buffer (1X HBSS + 5 mL FBS + 5 mL 0.5 M EDTA) and then surface stained with anti-mouse CD45 and anti-mouse EpCAM antibodies as above. Cells were then washed twice in column buffer and were then incubated at 37 °C with 5% CO2 for 20 min in pre-warmed complete RPMI media containing MitoSpy stain (25 mM). Cells were then washed twice in column buffer and then analyzed on a flow cytometer. Please see Supplementary Table 1 for a complete list of flow cytometry reagents.

### Bacterial colony-forming unit (CFU) enumeration

Fecal, ileal, and duodenal CFUs were enumerated by plating on BHI agar. Briefly, feces and SI luminal contents were collected from animals, were weighed, and then homogenized in sterile 1X HBSS buffer. Three serial ten-fold dilutions of homogenates were plated on BHI agar plates and incubated for 24 h under either aerobic or anaerobic conditions. CFU counts were standardized by content weight.

### Adoptive transfer experiments

Five-week-old CD19^−/−^ mice were randomly assigned to one of two treatment groups for B cell transfer experiments. Treatment groups received 10^7^ B cells isolated from the spleens of sex and age-matched WT or CD19^−/−^ donors via magnetic purification using the pan-B Cell Isolation Kit (STEMCELL). This kit obtains a cell purity of 98%. On day 0, B cells were administered via intraperitoneal injection and then animals were singly-housed for six weeks. At eleven weeks old (six weeks post transfer), animals were sacrificed and tissues were collected for analysis. B cell engraftment was determined by analyzing Peyer’s patches for CD19 + events in CD19^−/−^ recipients. Fecal pellets and mucus-associated bacteria were collected for 16S rRNA gene profiling. 15 cm of the small intestine were taken from mice. Approximately 5 cm of the ileum (immediately distal to the cecum) were collected for SI enteropathy scoring. The remaining small intestine tissue was processed as described above to isolate IECs for enumeration of the percentage of apoptotic IECs by flow cytometry. Ileal contents were also collected for CFU plating as described above.

### Quantitative PCR

qPCR was used to measure the abundance of *B.thetaiotamicron* in CD19^−/−^ mice colonized with isogenic *B.theta* strains (see description below). 40 days post-colonization, fecal pellets were collected, snap frozen in liquid nitrogen, and stored at −80 °C until use. Fecal DNA was extracted using the Allprep DNA/RNA Isolation Kit (with a 3 min bead beating step) (Qiagen). Sample DNA concentration was measured using a Nanodrop Spectrophotometer. Total bacteria were estimated using universal 16S qPCR primers (338F/518R), and *B.theta* abundance was estimated using *B.theta*-specific primers (Supplementary Fig. S13). Ct values were converted into amplicon copies using the following equation 10^((Ct-35)/−3)^. Amplicon copies were standardized to the amount (nanograms) of DNA added to each reaction.  qPCR was performed on a CFX Real-Time qPCR Instrument (Bio-Rad) using the iTaq Universal SYBR Green Supermix (Bio-Rad). Primer sets used in qPCR experiments are provided in Supplementary Table 2.

### RNAseq analysis of liver transcriptomes

Preparation of RNA and RNAseq analyses were carried out by the University of South Carolina Center for Targeted Therapeutics COBRE Functional Genomics Core. Total RNA was extracted from liver tissue using an RNeasy Mini Kit (QIAGEN). Briefly, liver tissues (~30 mg) were disrupted/homogenized in 2-Mercaptoethanol-containing RLT buffer using a TissueLyzer LT (QIAGEN) with 7 mm stainless steel beads for 5 min at 50 Hz. The lysate was transferred to a new tube, centrifuged at full speed for 3 min and the supernatant was carefully transferred to a new tube. Equal volume of 70% ethanol was added, mixed by pipetting, transferred to a RNeasy spin column, and spun down for 15 s at 15,000 × *g*. The column was washed by centrifugation at RT for 15 s at 15,000 × *g* with RW1 buffer, treated with DNase (QIAGEN) for 15 min at RT, washed with RW1 buffer, washed with RPE buffer twice, washed with 80% ethanol, and dried by centrifugation for 4 min at full speed. RNA was eluted into a new tube by adding 35 μL of Rnase-free water to the column and spinning it for 1 min at 15,000 × *g*. RNA purity and concentration was assessed using a NanoDrop 2000c spectrophotometer (Thermo Scientific). RNA integrity was determined using an Agilent 2100 Bioanalyzer (Agilent, Cat. No. G2939BA) where RNA Integrity Numbers ranged from 9.3 to 9.6 for all samples. RNA libraries were prepared using established protocol with NEBNExt Ultra II Directional Library Prep Kit (NEB, Lynn, MA). Each library was made with one of the TruSeq barcode index sequences and Illumina sequencing was performed by GENEWIZ, Inc. (South Plainfield, NJ) with Illumina HiSeq4000 (150 bp, pair-ended). Sequences were aligned to the *Mus Musculus* genome GRCm38.p5 (GCA_000001635.7, ensemble release-88) using STAR v2.4. Samtools (v1.2) was used to convert aligned sam files to bam files and reads were counted using the featureCounts function of the Subreads package with Gencode.vM19.basic.annotation.gtf annotation file. Only reads that were mapped uniquely to the genome were used for gene expression analysis with on average 87% ± 0.01% of all reads being assigned). On average 4% of reads were unassigned due to ambiguous base calling and 9% were unassigned due to non-gene alignment against the mouse genome. Differential expression analysis was performed in R using the edgeR package. The average read depth for the samples was 14,144,535 and only genes with at least one count per million average depth were considered for differential expression analysis. Raw counts were normalized using the Trimmed Mean of M-values (TMM) method. The normalized read counts were fitted to a quasi-likelihood negative binomial generalized log-linear model using the function glmQLFit. Genewise statistical tests for significant differential expression were conducted with empirical Bayes quasi-likelihood F-tests using the function glmQLFTest. Significant differentially expressed genes were included in the analysis that had an FDR *q*-value of <0.2.

### Enzymatic assay for ileal and fecal BA quantification

Fecal and ileal samples were collected from mice and frozen at −80 °C until use. On day of assay, 50 mg of materials were added to 1.5 mL microfuge tube and homogenized in 150 μL of ultra-pure water. Samples were vortexed for 30 s and allowed to settle at RT. Supernatants were then used for BA quantification using the Total Bile Acid Assay Kit (Sigma, cat#MAK309). This is a colorimetric assay where fluorescence is produced by the reactivity of 7α-hydroxysteroid dehydrogenase with mammalian BAs.

### BA mass spectrometry

Weighed fecal and SI samples were provided frozen in Precellys soft tissue homogenizing CK14 tubes (Bertin Technologies) to the Duke Proteomics Core for UPLC mass-spectrometry analysis (Supplementary Note 1). Extraction buffer was prepared by adding 6.8 mL ethanol to 0.96 mL water and 0.24 mL 100 mM pH 7.4 phosphate buffer. Three volumes of extraction buffer were added to each sample 3:1 volume:weight. Thus, all UPLC-MS BA quantifications were normalized to sample weight. Samples were then homogenized using three 10 s pulses in the Precellys Evolution between which samples were chilled using the Cryolys cooling system. The samples were then sonicated in an ice water bath for 5 min. The homogenized samples were stored at −80 °C until the day of extraction with the Bile Acids kit. On the day of bile acids sample extraction, the homogenized samples were thawed and vortexed. The samples were then centrifuged at 10,000 × rcf for 10 min in a refrigerated (4 °C) centrifuge and then stored on ice until addition to the bile acids kit plate. Samples were prepared using the Bile Acids kit (Biocrates) in strict accordance with their detailed protocol. Addition of 10 µL of the supplied internal standard solution to each well of the 96-well extraction plate was followed by drying under a gentle stream of nitrogen. Samples, blanks, calibration standards, and QCs were added in 10 µL aliquots to the appropriate wells. The plate was then dried under a gentle stream of nitrogen for 10 min. The samples were eluted with methanol then diluted with water for UPLC analysis. UPLC separation of bile acids was performed using a Waters Acquity UPLC (Milford, MA) with a proprietary reversed-phase UPLC column and guard column provided by Biocrates. Analytes were separated using a gradient from 10 mm ammonium acetate, 0.015% formic acid in water to 10 mm ammonium acetate, 0.015% formic acid in acetonitrile (65%), and methanol (30%). Total UPLC analysis time was approximately 6 min per sample. Using electrospray ionization in negative ion mode, samples were introduced directly into a Xevo TQ-S triple quadrupole mass spectrometer (Waters) operating in the Multiple Reaction Monitoring (MRM) mode. MRM transitions (compound-specific precursor to product ion transitions) for each analyte and internal standard were collected over the appropriate retention time. The UPLC-MS/MS data were imported into Waters application TargetLynx™ for peak integration, calibration, and concentration calculations. The UPLC-MS/MS data from TargetLynx™ were analyzed using Biocrates Me*tIDQ*™ software. Thus, all UPLC-MS BA quantifications were normalized to sample weight.

Supplementary Note 1: A more detailed description of UPLC-MS methods employed in this study and the raw output analysis performed by the Duke Proteomics Core without regard for experimental cohorts is provided in the “Supplementary Data, Tables, and Methods” document associated with this manuscript.

### *bsh* activity assay

Fecal pellets and ileal contents were shipped to the Patterson Lab at Pennsylvania State University for *bsh* activity measurements by mass spectrometry. Bacterial *bsh* activity was measured by mass spectrometry as previously described^69^. The total abundance of TCA-d4 and its’ deconjugation product (CA-d4) were quantified for each sample.

### Bacteroides thetaiotamicron colonization experiments

CD19^−/−^ mice were randomly assigned to one of two treatment groups for colonization experiments. Each group of animals was colonized with one of two isogenic strains of *B.thetaiotamicron* (hereafter *B.theta*). WT *B.theta* (strain #1) and an isogenic strain that lacks a functional *bsh* gene (Δ*bsh-B.theta* (strain #2)) were kindly provided by Dr. Sloan Devlin (Harvard University). *B.theta* strains were grown in BHI media under anaerobic conditions. Prior to colonization (Day 0), CD19^−/−^ mice were given *ad libitum* access to antibiotic-treated drinking water containing (0.5 mg/mL) metronidazole and (0.5 mg/mL) ciprofloxacin for one week to reduce commensal bacteria abundance. For colonization, antibiotic water was removed for 48 h (Day 8 and 9) and animals were administered bacteria (Day 10). Animals were colonized with ca. 10^8^ *B.theta* CFUs from log-phase liquid culture via oral gavage. Mice were then individually housed for 30 days. At day 30 post-colonization, animals were sacrificed and tissues were collected. Ileal contents were collected for quantification of BAs by mass-spectrometry.

### 16S microbiota profiling

Fecal pellets and ileal contents (both ~50 mg) were collected from animals and placed into 2 mL collection tubes containing sterile garnet stones for downstream bead-beating and DNA isolation following AllPrep Dual DNA/RNA Isolation Kit instructions (QIAGEN). Briefly, for fecal pellet collection, mice were scruffed and fecal pellets were collected directly into bead-beater tubes. For ileal content collection, mice were then euthanized and the ileum was removed using sterile forceps and scissors. Forceps were then used to squeeze luminal contents from the ileum into collection tubes. Collection of ileal contents differed between 16S experiments. For comparison of WT and CD19^−/−^ mouse ileal microbiota, the entire contents of the ileum were placed in a 1.5 mL microfuge tube and mixed. Approximately 50 mg of mixed materials were then placed in bead-beater tube for DNA isolation. For comparison of ileal microbiota in CD19^−/−^ adoptive transfer recipients, contents in the distal 3 cm of the ileum of mice were squeezed directly into a bead-beater microfuge tube for DNA isolation. All DNA extractions were carried out in a biosafety cabinet using strict aseptic technique. Animals were chosen for inclusion in sequencing experiments using the following criteria: (1) both males and females from each genotype were included in analysis, (2) animals were derived from multiple breeding cages from each genotype, (3) mice were sampled from several different stock cages for each genotype, (4) mice were derived from different litters from the same breeder pair, (5) the age-range of mice (eight to 13 weeks old) was similar between genotypes. Isolated DNA was submitted to the University of Alabama at Birmingham Heflin Center Genomics Core for 16S sequencing on an Illumina MiSeq. Raw fastq reads were de-multiplexed and forward and reverse primer sequences were trimmed from reads. This yielded a 251 bp product spanning the V3/V4 region of the bacterial 16S rRNA gene. All 16S analyses were carried out using QIIME 2.0 analysis pipeline^70,71^. De-multiplexed fastq files were imported as a QIIME 2.0 artifact for downstream processing and analysis. De-multiplexed reads were trimmed (forward 251 bp; reverse 251 bp) based on quality score cutoff of Q-score<15. Denoising was performed using DADA2 with amplicon sequence variant (ASV) taxonomic calls being made against the GreenGenes database (13.8). Low quality reads and chimeras were filtered out of final feature table using DADA2. Prior to beta and alpha diversity analyses, feature tables were rarified to even sampling depth (WT versus CD19 ileal community comparison: rarefaction depth=54,312 (Supplementary Fig. S14A); B cell transfer experiment: fecal rarefaction depth=24003, ileal rarefaction depth=28688) (Supplementary Fig. S14B). Based on Bray–Curtis analysis of our WT versus CD19^−/−^ ileal data, sex (PERMANOVA, pseudo-Fstat_2,29_ = 2.38, *p* = 0.03), and age (PERMANOVA, pseudo-Fstat_5,29_ = 1.95, *p* = 0.02) have significant effects on microbiota composition, whereas cage does not (PERMANOVA, pseudo-Fstat_11,29_ = , *p* = 0.06). Bray–Curtis PcoA plots showing balanced variability between genotypes in sex, age, and cage sampling are provided (Supplementary Fig. S14C). This data also demonstrates that sex and age do not explain observed differences between genotypes. The results of 16S rRNA microbiota profiling experiment were used to infer microbial gene abundance using the PICRUSt2.0 plug-in in QIIME2.0^72^. Briefly, rarified ASV tables were used to identify microbial genes with gene calls being made against the KEGG gene database. This table was used to calculate *bsh* abundance in WT and CD19^−/−^ mice. Random Forest Analysis was performed using Qiime 2.0. Lefse analysis was performed using default parameter settings within the Galaxy pipeline^73^.

### Histology

Approximately 5 cm of the distal ileum (proximally from the ileo-cecal valve) were collected from euthanized animals and placed immediately in 10% buffered formalin. Tissues were submitted for processing to the USC Instrumentation Resource Facility Histology Core. Paraffin block sections were stained with hematoxylin and eosin (H&E) and subsequently analyzed blindly by a certified pathologist and characterized according to the presence of histo-morphological changes of inflammation. For the evaluation of the specimens of the distal ileum, the scoring system suggested by Erben et al.^74^ was applied and the parameters evaluated included inflammatory infiltrate (severity and extent), as well as epithelial (crypt hyperplasia) and mucosal (villous blunting) changes of the intestinal wall. At least 4–5 consecutive villi from base to tip were rated. Tissue sections were examined and images were obtained by standard light microscopy using a Leica optical microscope system.

### Statistical analysis

Data sets for all experiments represent pooled data from two to three experimental replicates (*n* = 2–5 animals per genotype/treatment in each replicate). All of the reported data-points represent biological replicates. To enhance statistical rigor in our adoptive transfer and *B.theta* colonization experiments, animals were randomly assigned to treatment groups, were experimentally manipulated, and were then individually housed for the duration of the respective experiments. All univariate and bivariate statistical analyses were performed using PRISM8.0 Statistical Analysis Software (Graphpad). For all univariate statistical analyses, the normality of data was first determined using a Shapiro–Wilk’s test. In the case of non-normally distributed datasets, data was log-transformed and statistical testing was subsequently performed on log-transformed datasets. In the case of log-transformed normally-distributed datasets that failed to conform to the assumption of homoscedasticity, a Welch’s correction was applied to adjust *p*-values accordingly. For data that failed to conform to the assumption of normality after log-transformation, a two-tailed unpaired a non-parametric Mann–Whitney U test was applied to untransformed data. Because individual disease scores reflect ordinal data, a non-parametric Mann–Whitney U test was used for comparisons of these datasets. For bivariate analyses, a two-tailed one-way ANOVA was used to determine the significance of correlations with F-statistics and sample sizes shown for all analyses. All univariate and bivariate statistical analyses were performed using PRISM8.0 Statistical Analysis Software (Graphpad). In Figs. 1A, B, and 6C, outlier analysis was performed using the ROUT method (*Q* = 1%) and outliers were removed prior to statistical analysis. Multiple hypothesis testing *p*-value corrections using the Tukey’s or Sidak’s methods were used when appropriate and is indicated in figure captions. For qualitative descriptions of relationships between biological variables, heatmaps were constructed in PRISM8.0 and were based on Pearson r-coefficients and *R*^2^ values calculated in Microsoft Excel. For all pairwise comparisons, the mean is shown as the measure of central tendency. Error bars reflect ±standard error of the mean. For multivariate statistical analysis of 16S data (i.e., comparison of β-diversity) a PERMANOVA (999 iterations) was used to test for significant differences in β-diversity between genotypes. For RNAseq data, gene-wise statistical tests for significant differential expression (i.e., FDR-adjusted *p*-value cutoff of <0.05) were conducted with empirical Bayes quasi-likelihood F-tests using the function glmQLFTest.

### Reporting summary

Further information on research design is available in the Nature Research Reporting Summary linked to this article.

## Supplementary information

Supplementary Information

Description of Additional Supplementary Files

Supplementary Data 1

Supplementary Data 2

Supplementary Data 3

Supplementary Data 4

Supplementary Data 5

Supplementary Data 6

Reporting Summary

## Data Availability

Source data are associated with this paper. Source data for datasets summarized in this manuscript have been deposited and made publicly available through the Dryad Data Repository (10.5061/dryad.rxwdbrv9h). Raw sequence data and relevant metadata for all 16S analyses shown in this manuscript have been deposited in the NCBI short read archive (SRA) under Bioproject ID#PRJNA773874. Raw sequence data and relevant metadata for ileal and liver RNAseq datasets have been deposited into the NCBI Gene Expression Omnibus (GEO) under the GEO identifiers GSE186435 and GSE186436, respectively. Source data are provided with this paper.

## Source data

Source Data
