# Supplementary material for: Defective humoral immunity disrupts bile acid homeostasis which promotes inflammatory disease of the small bowel
Source: Nat Commun. 2022 Jan 26;13:525. doi: 10.1038/s41467-022-28126-w (PMC8792037; doi:10.1038/s41467-022-28126-w)
Supplement: Supplementary file 1 — Supplementary Information [file 41467_2022_28126_MOESM1_ESM.pdf]

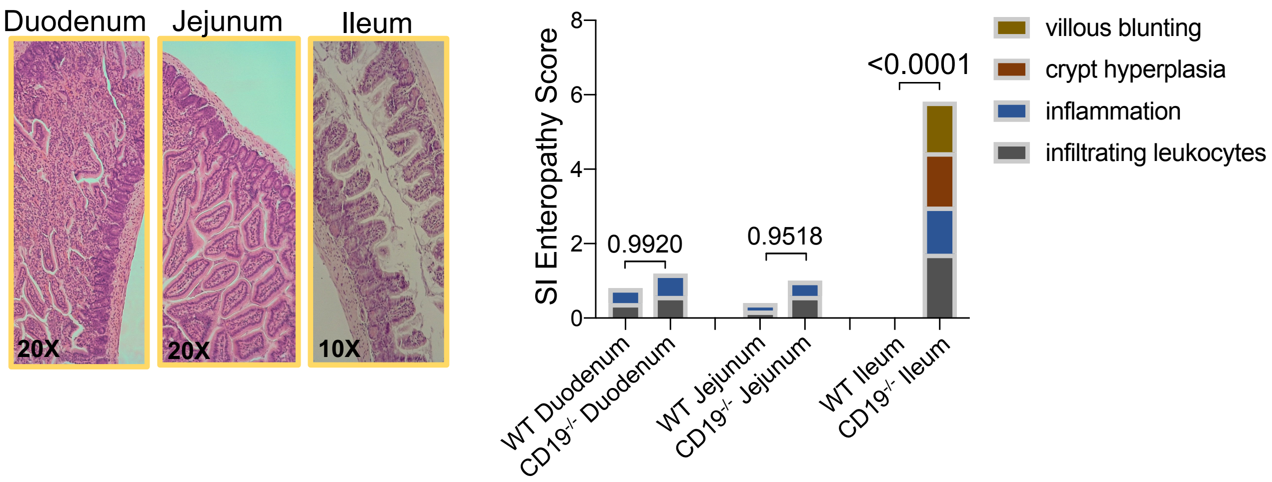

Supplementary Figure S1. SI enteropathy is restricted to the ileum of CD19$^{-/-}$ mice. Duodenal (n=5 WT, n=5 CD19$^{-/-}$), jejunal (n=5 WT, n=5 CD19$^{-/-}$), and ileal (n=6 WT, n=6 CD19$^{-/-}$) sections were generated from WT and CD19$^{-/-}$ mice and H&E stained. SI enteropathy was scored using the four-point scoring method described in Materials and Methods. Two-tailed ANOVA with multiple comparisons (a Tukey's correction for multiple comparisons was applied); ns=non-significant, ****=p<0.0001.

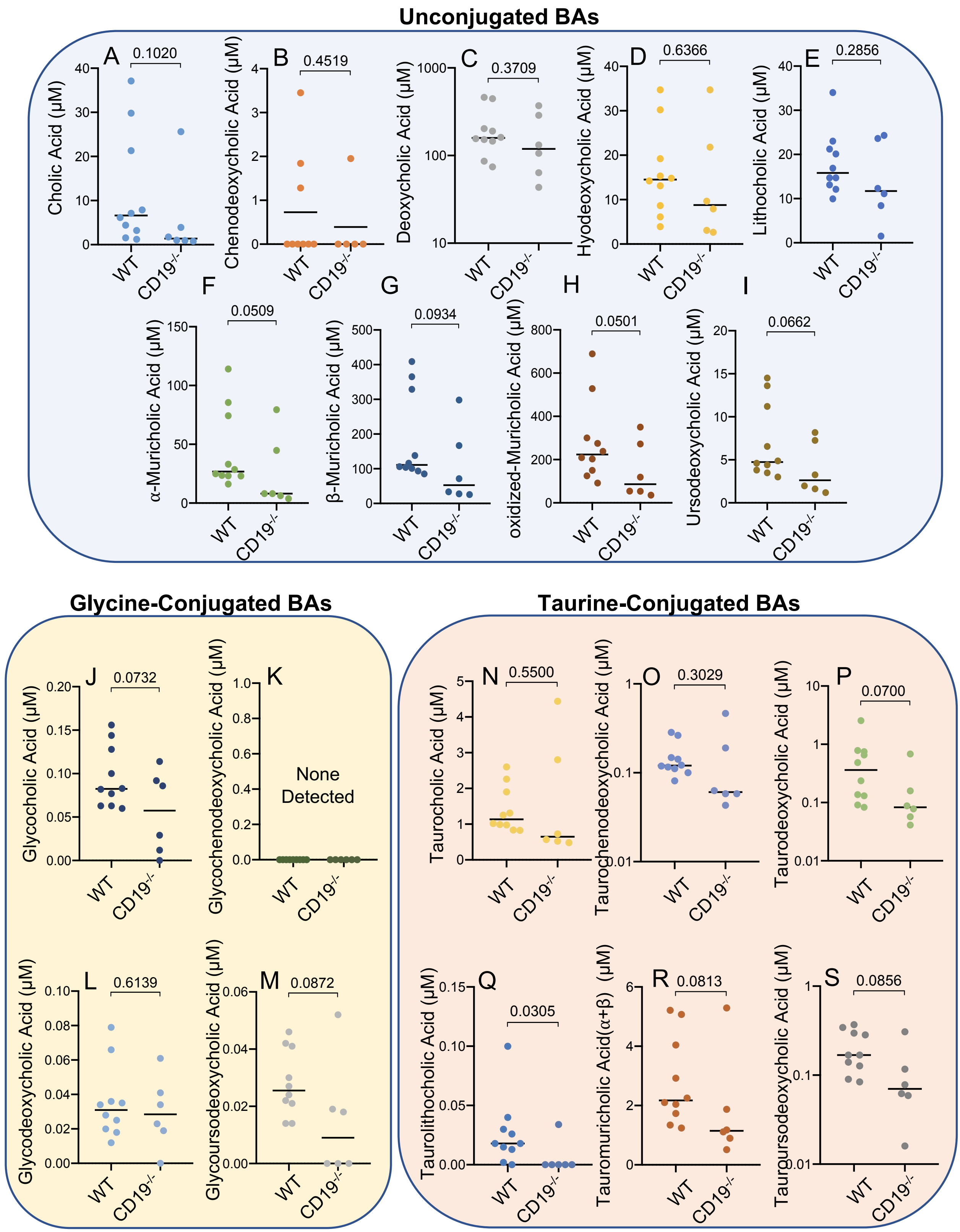

Supplementary Figure S2. Pairwise comparison of nineteen fecal BAs between WT and CD19$^{-/-}$ mice. Measured by UPLC-MS and shown as μM concentrations. **(B, G, M, Q)** Two-tailed unpaired Mann Whitney U test. **(A, C, D, E, F, H, I, J, L, N, O, P, R, S)** Two-tailed unpaired Student's t-test. Each comparison stands alone, no correction for multiple hypothesis testing.

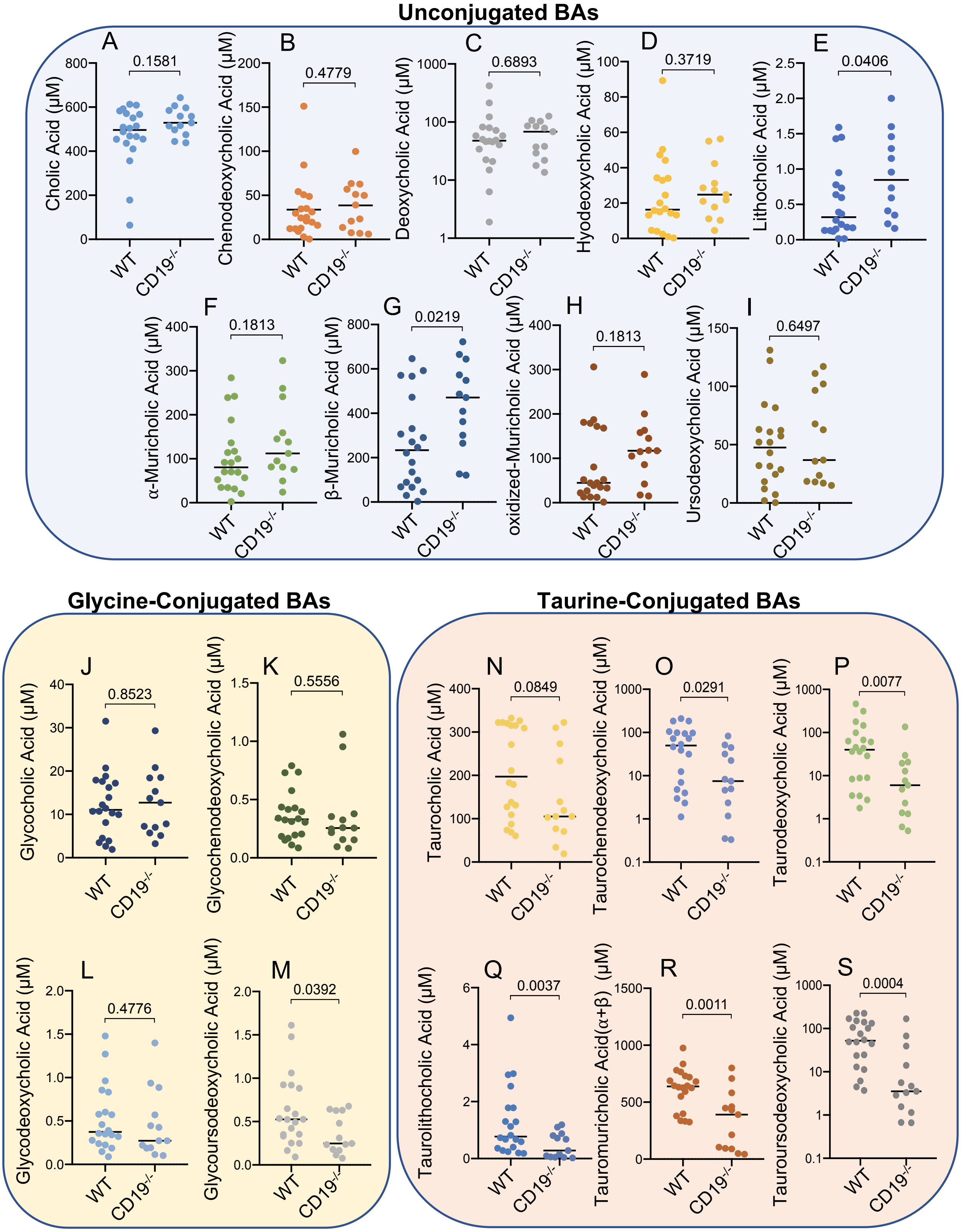

Supplementary Figure S3. Pairwise comparison of nineteen BAs measured in SI contents of WT and CD19$^{-/-}$ mice. Measured by UPLC-MS and shown as µM concentrations. **(A, B, D, F, G, H, I, N, O)** Two-tailed unpaired Mann Whitney U test. **(C, E, J, K, L, M, P, Q, R, S)** Two-tailed unpaired Student's t-test. Each comparison stands alone, no correction for multiple hypothesis testing.

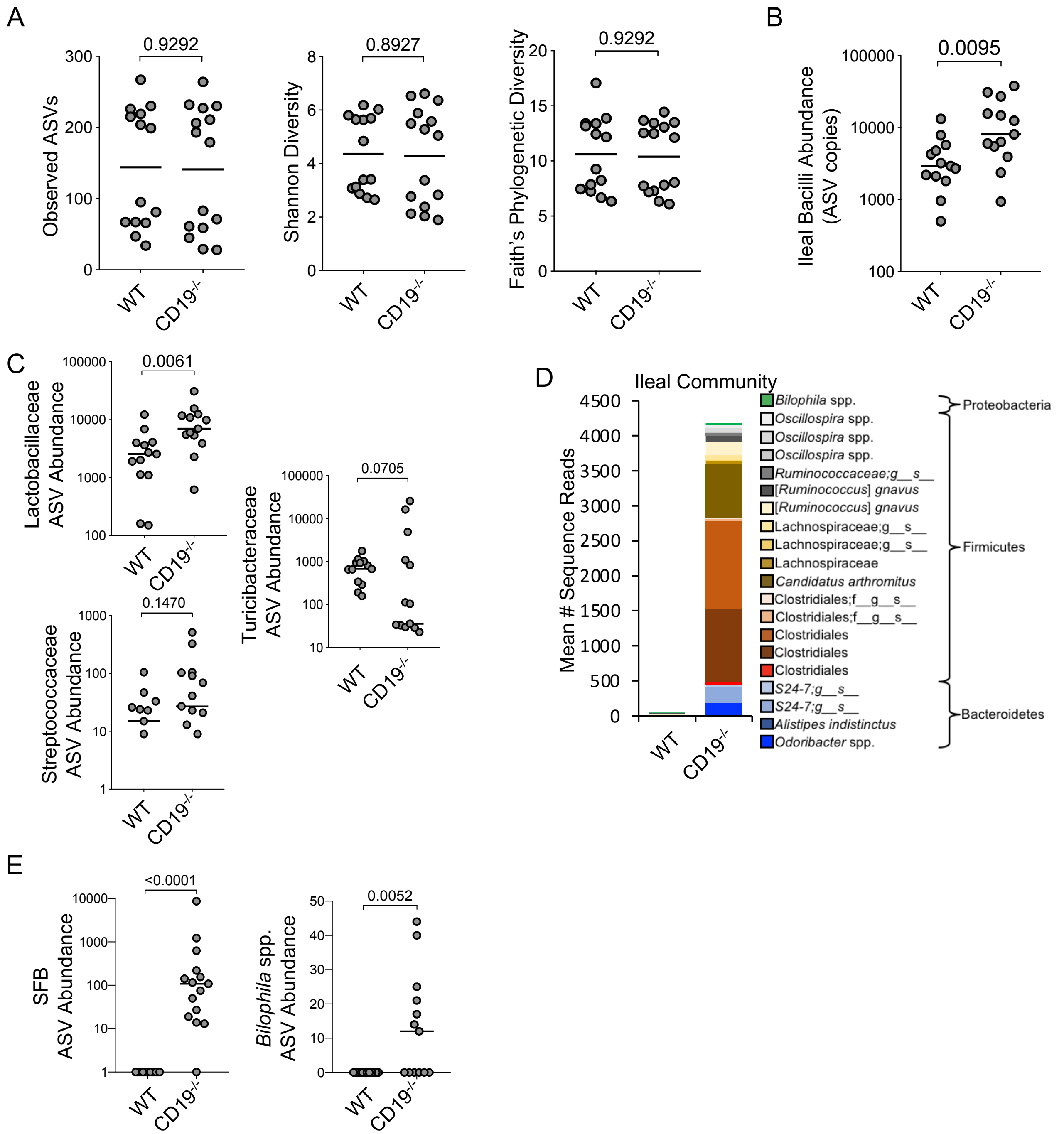

Supplementary Figure S4. Microbiota compositional shifts in CD19$^{-/-}$ mice. (A) Comparison of α-diversity metrics between WT and CD19$^{-/-}$ mice is shown. **(B)** The relative abundance of Bacilli in WT and CD19$^{-/-}$ mice is shown. **(C)** Bacilli ASVs (driven primarily by expansion of members of the Lactobacillaceae family) are outgrown in CD19$^{-/-}$ mice. **(D)** CD19$^{-/-}$ have outgrowth of specific ASVs that are predominantly members of the Class Clostridia. (A, B, C, E) Two-tailed unpaired Mann-Whitney U tests. (D) Stacked bar-plots represent relative abundance of bacterial groups whose abundance was determined to be statistically significantly different based on Discrete False Discovery (DS-FDR) hypothesis testing. (A-E) Data in all plots representative of two replicate experiments.

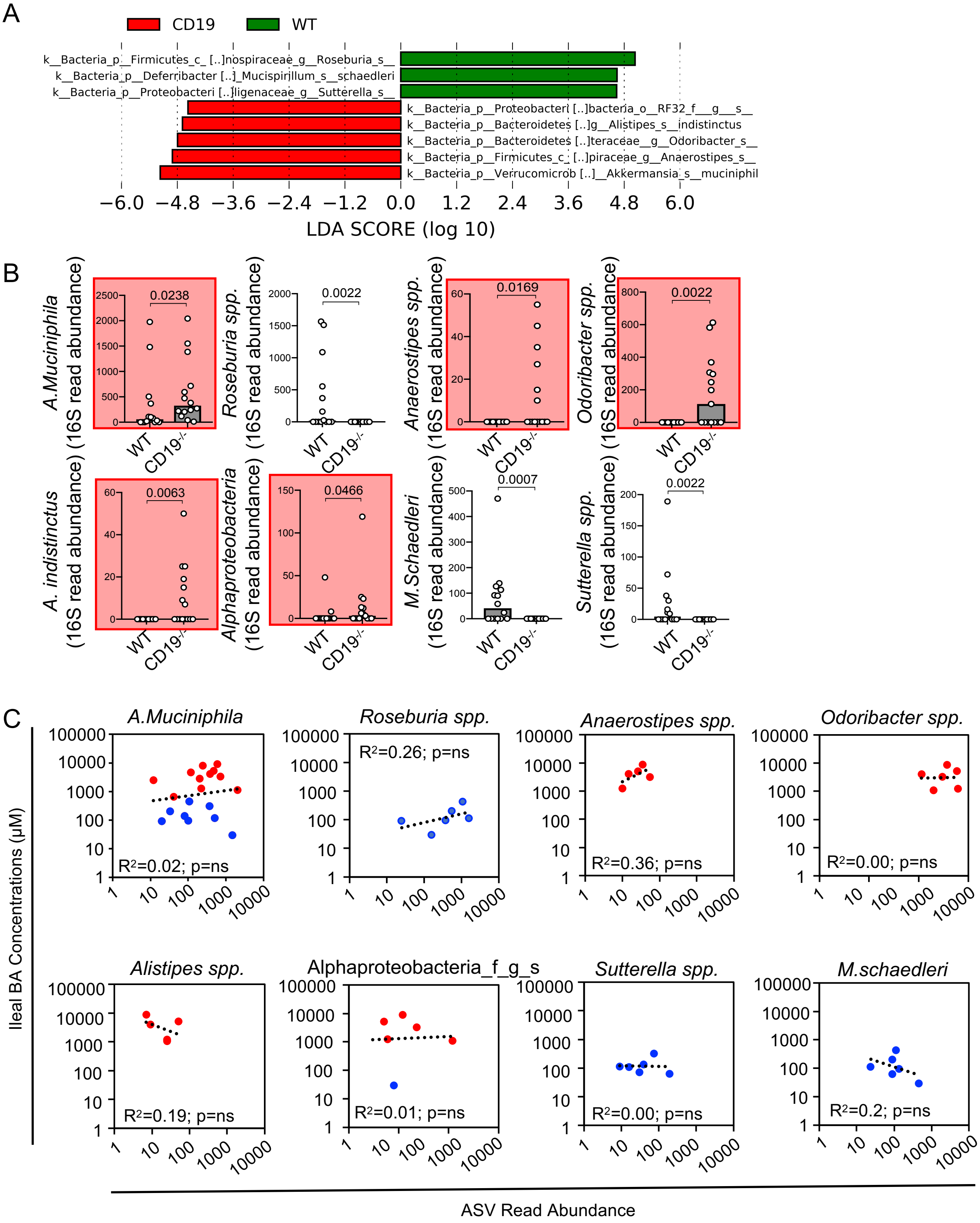

Supplementary Figure S5. Results of Lefse/BA concentration analysis. (A) Lefse analysis identified seven taxa that were significantly differentially enriched between WT and CD19$^{-/-}$ mice by linear discriminant analysis (LDA). LDA plot of Lefse analysis is shown. **(B)** Pairwise comparisons of read abundance for eight species identified as differentially enriched by Lefse. P-values derived from independent two-tailed unpaired Mann-Whitney U tests. Red boxes indicate anaerobic/facultative anaerobic bacteria. **(C)** Correlation analysis between ASV read abundance and ileal BA concentrations are shown. P-values shown are results of independent two-tailed one-way ANOVAs performed for each relationship.

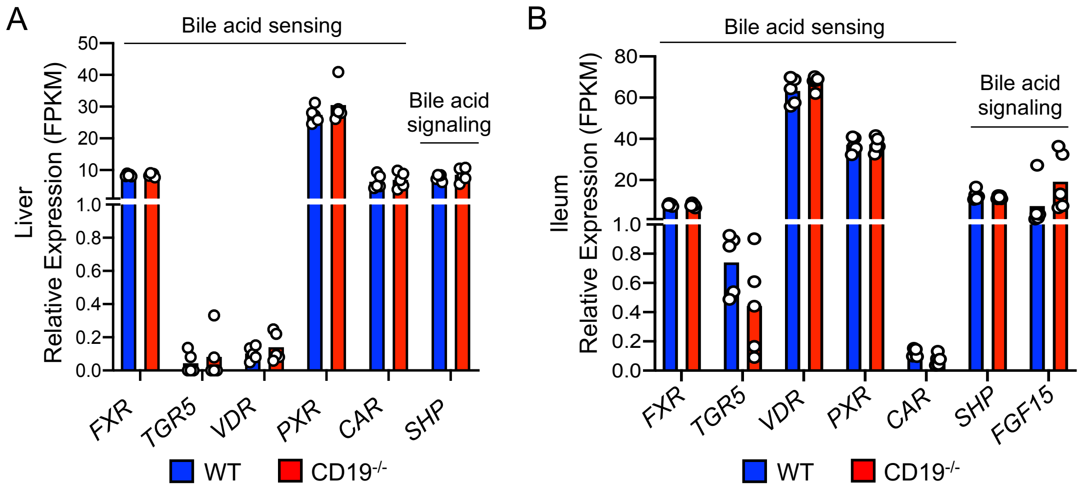

Supplementary Figure S6. BA receptor and signaling molecule expression is not perturbed in CD19$^{-/-}$ mice. (A) Comparison of read abundance (FPKM) of relevant liver BA receptors and downstream signaling molecules (*SHP*) between WT and CD19$^{-/-}$ mice is shown. **(B)** Comparison of read abundance (FPKM) of relevant ileal BA receptors and downstream signaling molecules (*SHP* and *FGF15*) between WT and CD19$^{-/-}$ mice is shown. (A and B) None of the genes shown are differentially regulated based on FDR significance cutoff of 0.05. Statistical results of quasi-likelihood hypothesis testing is provided in Supplementary Table T4 and Supplementary Table T6.

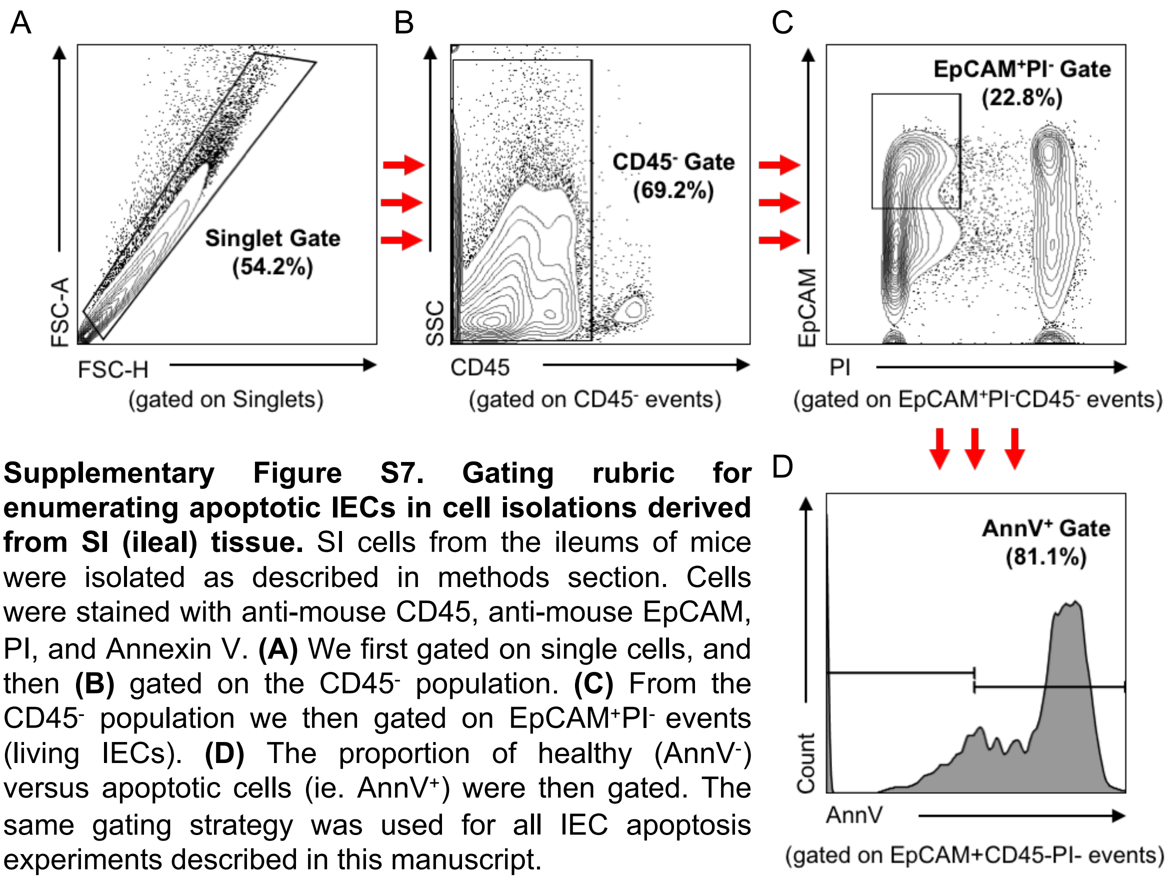

Supplementary Figure S7. Gating rubric for enumerating apoptotic IECs in cell isolations derived from SI (ileal) tissue. SI cells from the ileums of mice were isolated as described in methods section. Cells were stained with anti-mouse CD45, anti-mouse EpCAM, PI, and Annexin V. **(A)** We first gated on single cells, and then **(B)** gated on the $CD45^-$ population. **(C)** From the $CD45^-$ population we then gated on $EpCAM^+PI^-$ events (living IECs). **(D)** The proportion of healthy ($AnnV^-$) versus apoptotic cells (ie. $AnnV^+$) were then gated. The same gating strategy was used for all IEC apoptosis experiments described in this manuscript.

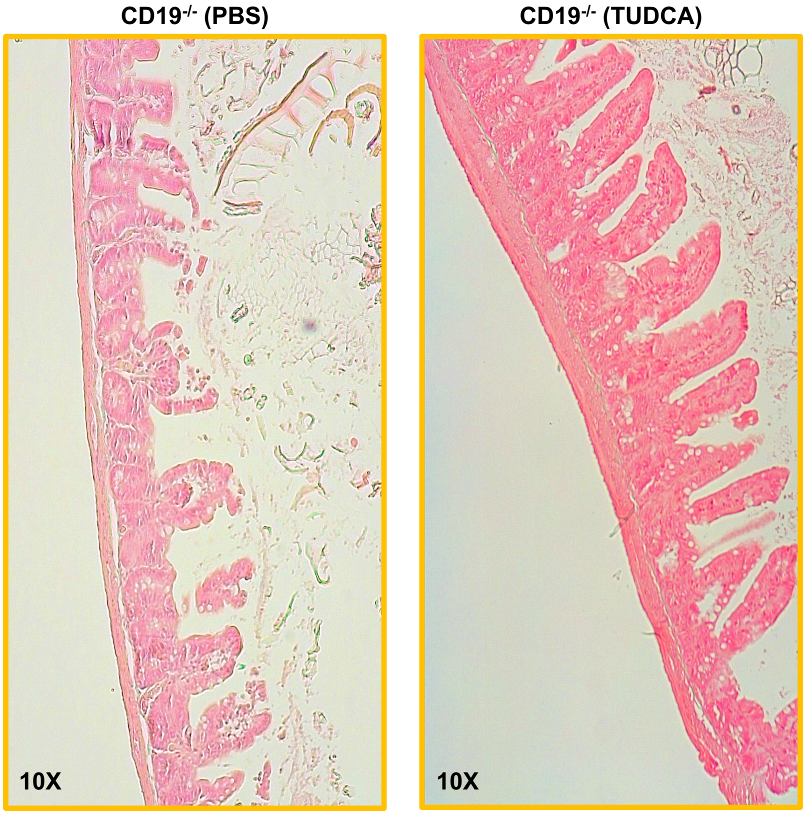

Supplementary Figure S8. Histological comparison of ileal sections derived from CD19$^{-/-}$ mice treated with vehicle (1XPBS) or TUDCA. Representative H&E stained sections of ileum (10X magnification) are shown for comparison. Images are representative of the degree of SI enteropathy observed in PBS- (n=10) and TUDCA-treated (n=9) CD19$^{-/-}$ mice across three replicate experiments.

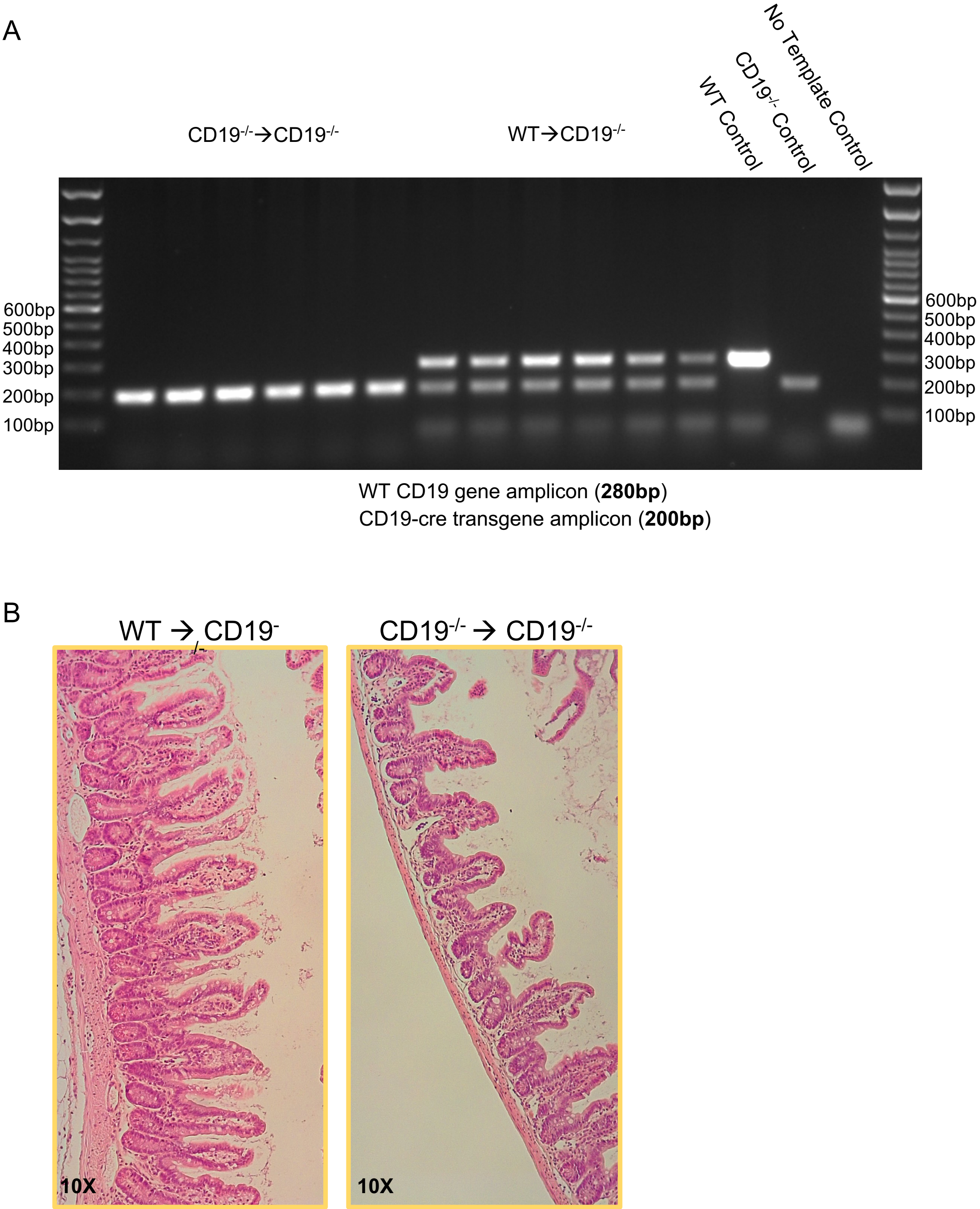

Supplementary Figure S9. B cell transfer validation and histological comparison of ileal sections derived from $CD19^{-/-}$ mice receiving 10^7 B cells from either WT (WT→$CD19^{-/-}$) or $CD19^{-/-}$ ($CD19^{-/-}$→$CD19^{-/-}$) donors. (A) Results of PCR detection of WT CD19 allele and CD19-cre transgene in $CD19^{-/-}$ mice receiving WT B cells (n=6 shown) or $CD19^{-/-}$ B cells (n=7 shown). DNA was extracted from ileal tissues of $CD19^{-/-}$ mice six weeks post B cell transfer and PCR was conducted to detect the presence of WT CD19 sequence amplicons, which indicates successful engraftment and homing of WT B cells to the gut of $CD19^{-/-}$ recipients. **(B)** Representative H&E stained sections of ileum (10X magnification) from $CD19^{-/-}$ mice receiving 10^7 B cells from WT (n=8) or $CD19^{-/-}$ donors (n=9) are shown. (a and B) Data is representative of the degree of SI enteropathy observed between experimental groups across two replicate experiments.

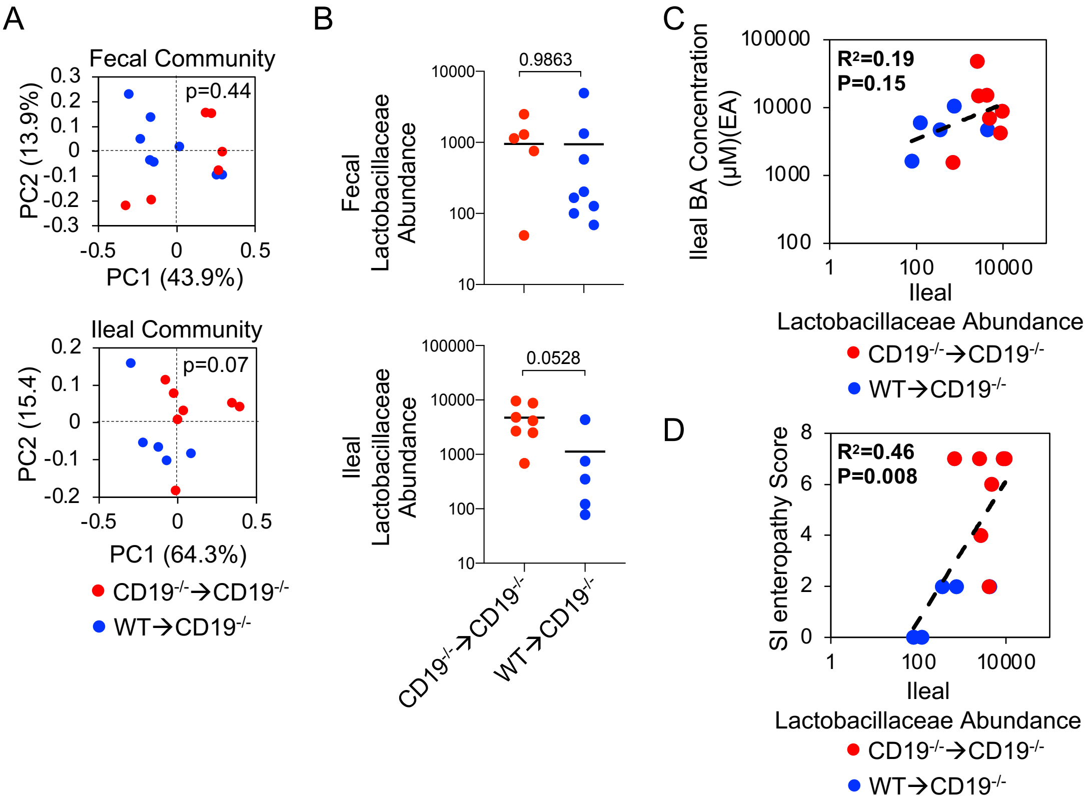

Supplementary Figure S10. Results of 16S analyses of B cell transfer experiments. (A) PcoA plots based on Bray-Curtis analysis of β-diversity between $CD19^{-/-}$ mice receiving WT B cells (WT→$CD19^{-/-}$) or $CD19^{-/-}$ B cells ($CD19^{-/-}$→$CD19^{-/-}$). Fecal community: PERMANOVA, pseudo-$Fstat_{2,14}$=0.91, p=0.44. Ileal community: PERMANOVA, pseudo-$Fstat_{2,12}$=2.75, p=0.07. **(B)** Pairwise comparisons of Lactobacillaceae abundance between adoptive transfer cohorts are shown. Two-tailed unpaired Students t-test. **(C)** The correlation between ileal Lactobacillaceae abundance and ileal BA concentration is shown. Two-tailed one-way ANOVA, $Fstat_{1,10}$=2.40, p=0.15. **(D)** Correlation analysis of ileal Lactobacillaceae abundance and SI enteropathy score is shown. Two-tailed one-way ANOVA, $Fstat_{1,10}$=10.81, p=0.008.

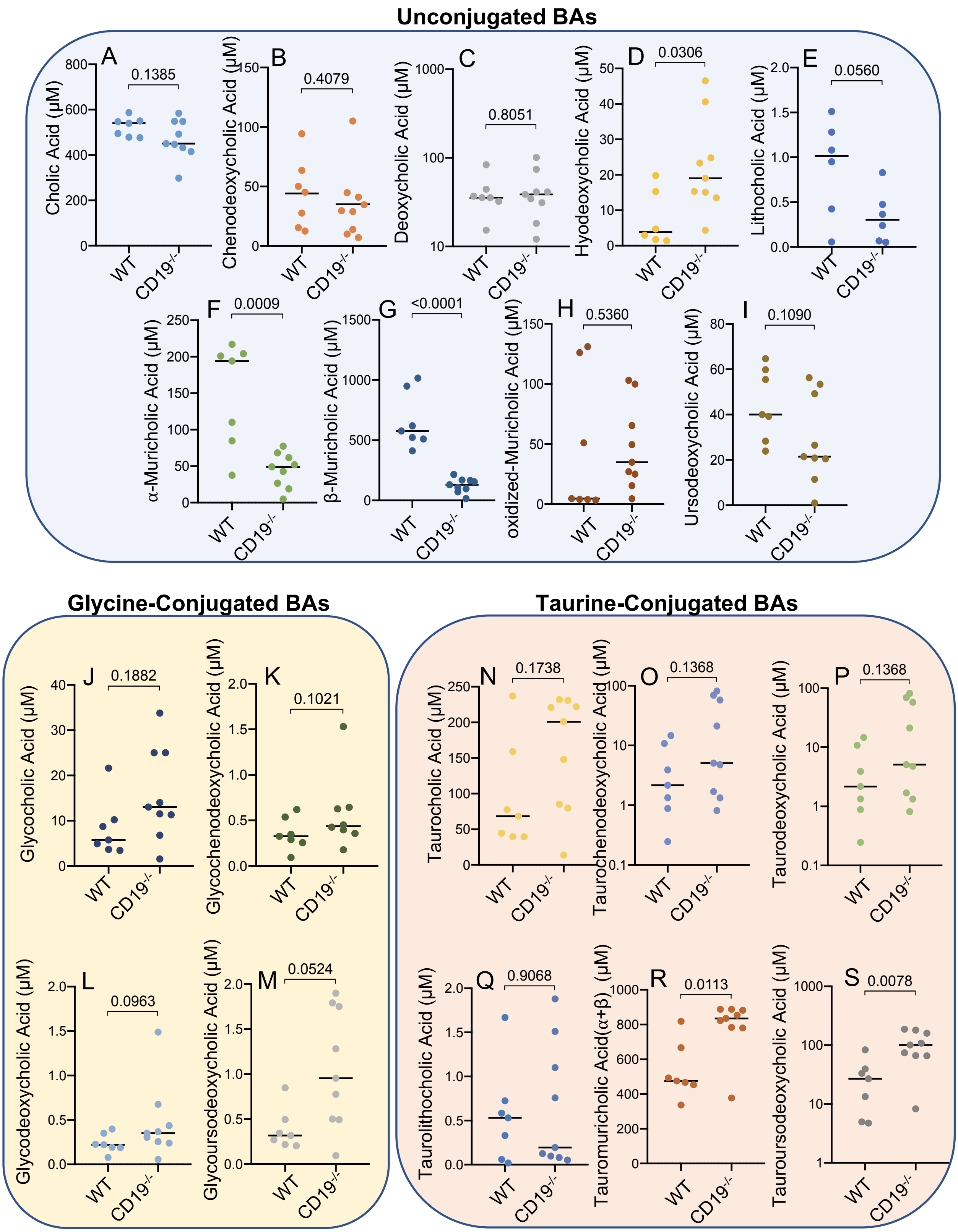

Supplementary Figure S11. Pairwise comparison of nineteen ileal BAs between CD19$^{-/-}$ mice colonized with WT B.theta or *Δ*bsh B.theta. Measured by UPLC-MS and shown as μM concentrations. **(B, H, L, N, R)** Two-tailed unpaired Mann Whitney U test. **(A, C, D, E, F, G, I, J, K, M, O, P, Q, S)** Two-tailed unpaired Student's t-test. Each comparison stands alone, no correction for multiple hypothesis testing.

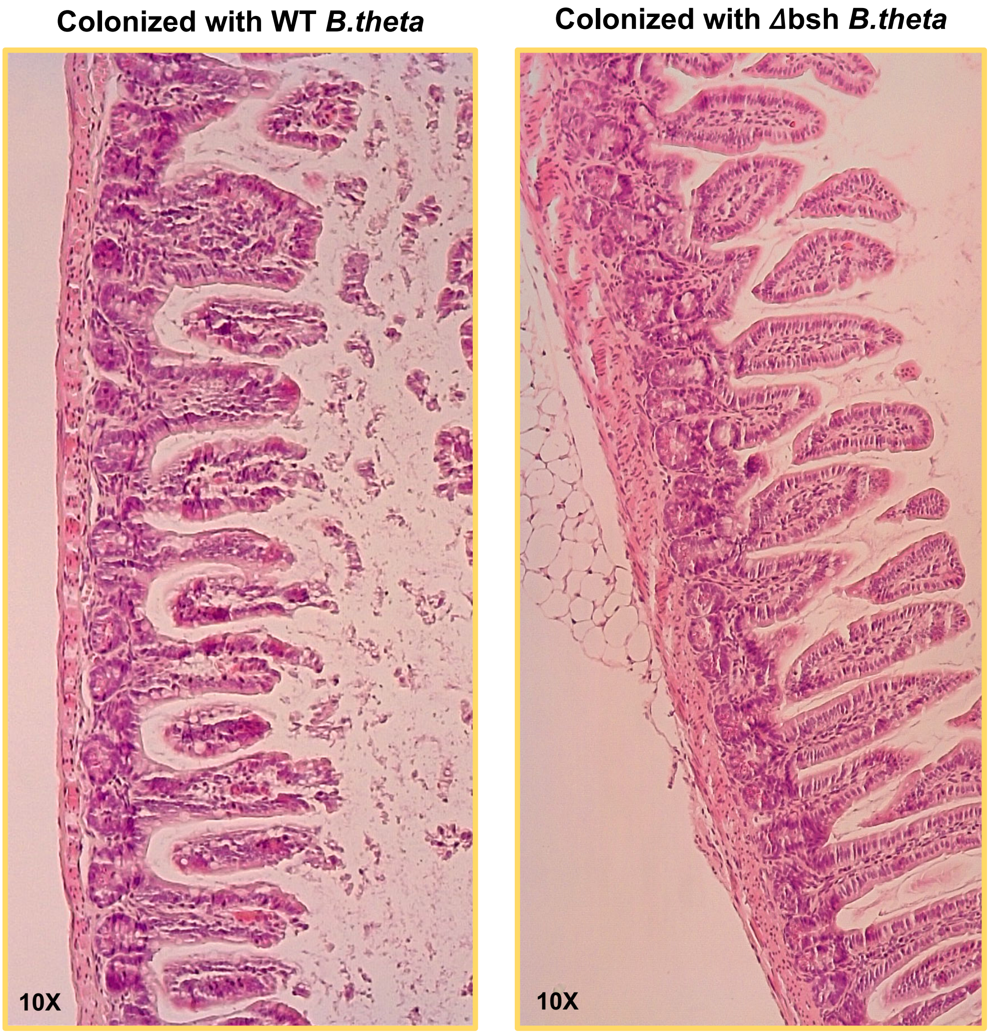

Supplementary Figure S12. Histological comparison of ileal sections derived from CD19$^{-/-}$ mice colonized with WT *B.theta* or Δbsh *B.theta*. Representative H&E stained sections of ileum (10X magnification) are shown for comparison. Images are representative of the degree of SI enteropathy observed in CD19$^{-/-}$ mice colonized with WT *B.theta* (n=7) or *Δbsh B.theta* (n=8). Data representative of 2 replicate experiments.

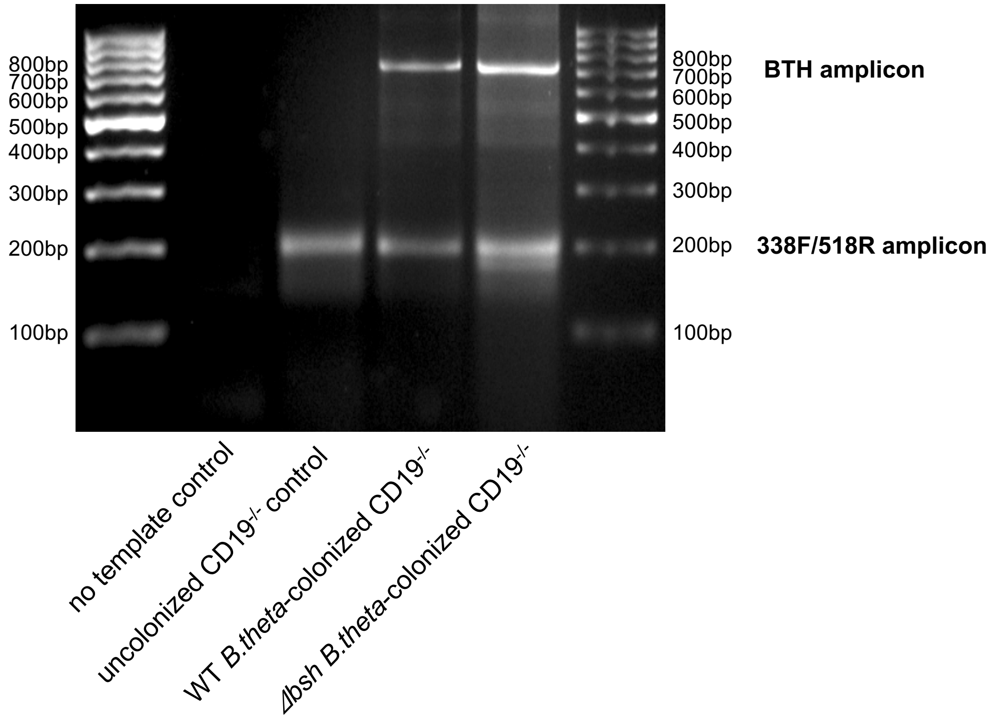

Supplementary Figure S13. Control gel depicting amplicon band size generated during qPCR assay used to determine the abundance of *Bacteroides thetaiotamicron* (both *WT B.theta* and *Δbsh B.theta* isogenic strains) and total Bacteria in colonized mice. The universal bacterial primer set 338F/518R generates an approximately 200bp amplicon and the *B.theta*-specific BTH primer set generates a 721bp amplicon. One mouse from each of the three conditions above were used to confirm expected band sizes for each amplicon.

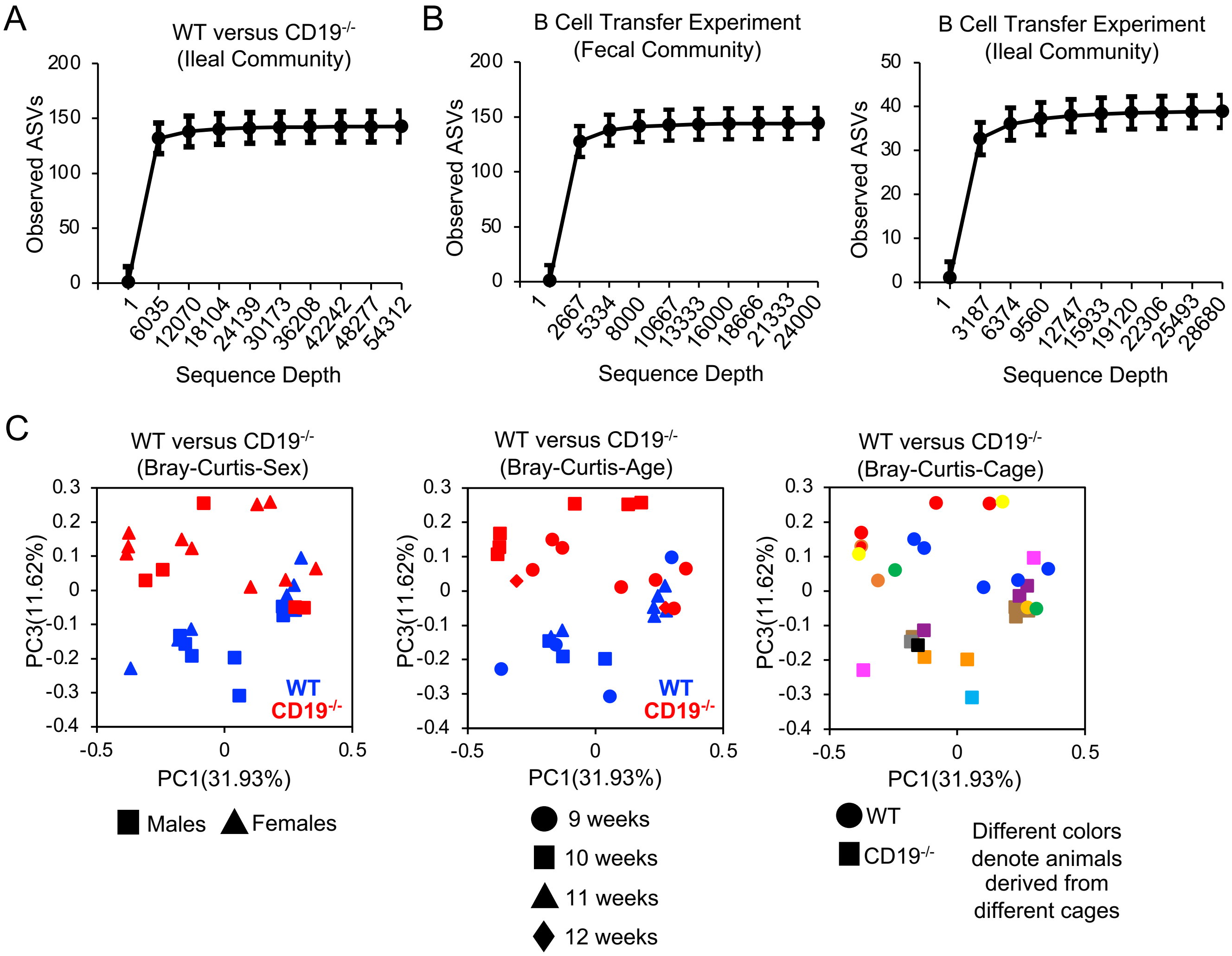

Supplementary Figure S14. Metrics of microbiota sequencing experiments. (A) Alpha-rarefaction plot demonstrating ASV diversity sampling as a function of sequence depth in the 16S microbiota profiling experiment comparing ileal microbial communities of WT and CD19$^{-/-}$ mice (n=29 mice). **(B)** Alpha-rarefaction plot demonstrating ASV diversity sampling as a function of sequence depth in the fecal (n=8 WT and n=6 KO mice) and ileal (n=5 WT and n=7 KO) datasets from CD19$^{-/-}$ B cell transfer 16S microbiota profiling experiments. **(C)** PcoA plots of Bray-Curtis estimates of β-diversity showing equal variation in sampling between genotypes. Colors in far right plot denote different breeding cages animals were derived from for use in experiments. Sex, age, and source cage do not explain observed differences between genotypes. (A-B) Data are presented as mean values +/- SEM .

Supplementary Table 1. List of flow cytometry antibodies and solutions used in this study.

Antibody	Fluor	Clone	Vendor	Catalog #	Concentration
Anti-mouse CD45	APC.Cy7	30-F11	BioLegend	103113	0.2 mg/ml
Anti-mouse CD45	APC	30-F11	BioLegend	103115	0.2 mg/ml
Anti-mouse CD326	Alexa 488	G8.8	BioLegend	118210	0.5mg/ml
Anti-mouse CD326	PE.Cy7	G8.8	BioLegend	118215	0.2mg/ml
Anti-mouse Ki-67	APC	16A8	BioLegend	652405	0.2mg/ml
Annexin V	APC	-	BioLegend	640920	8μg/ml
Propidium Iodide	-	-	BioLegend	421301	0.5mg/ml
Zombie Green	FITC	-	BioLegend	423111	-
MitoSpy™NIR DiIC1(5)	APC	-	BioLegend	424807	-

Supplementary Table 2. List of primers used in this study.

Gene	Primer Sequence	Reference
All Bacteria	(EUB-338f)(forward) 5'-ACTCCTACGGGAGGCAGCAG-3'<br>(EUB-518r)(reverse) 5'-ATTACCGCGGCTGCTGG-3'	1
B.thetaiotamicron	(BTH-F) 5'-TGGAGTTTTACTTTGAATGGAC-3'<br>(BTH-R) 5'-CTGCCCTTTTACAATGGG-3'	2

Supplemental References

1 Luo, G. *et al.* Historical Nitrogen Deposition and Straw Addition Facilitate the Resistance of Soil Multifunctionality to Drying-Wetting Cycles. *Applied and environmental microbiology* **85**, doi:10.1128/AEM.02251-18 (2019).

2 Teng, L. J. *et al.* PCR assay for species-specific identification of Bacteroides thetaiotaomicron. *J Clin Microbiol* **38**, 1672-1675 (2000).

Supplementary Note 1: UPLC-MS Methods

UPLC/MS-MS Analysis of Mouse Ileal Content Samples Using the Biocrates Bile Acids Kit

In collaboration with Dr. Jason Kubinak,

University of South Carolina School of Medicine

December 11, 2019

Objective: Measure the levels of selected bile acids in 40 mouse ileal content samples using the Biocrates Bile Acids kit.

Duke Proteomics and Metabolomics Core Facility Contributors: Lisa St. John-Williams (sample preparation, data collection, data analysis, report writing), Will Thompson (study design, scientific oversight, report writing), and Arthur Moseley (scientific oversight, report writing).

Introduction:

The Biocrates Bile Acids assay quantifies 20 bile acids, 16 of which are normally detected in human plasma. Three of the bile acids measured are murine-specific (MCA(a), MCA(b), MCA(o)). The bile acids kit includes all requisite calibration standards, internal standards, and QC samples. The use of these standards according to the detailed analysis protocol, which was validated in Biocrates' lab in Austria, assures assay harmonization and standardization within a project, across projects, and across laboratories. Sample analysis of bile acids are performed by a UPLC (ultra-high pressure liquid chromatography) tandem mass spectrometric method using a reversed phase analytical column for analyte separation (LC/MS-MS, **Figure 1**). Selective analyte detection is accomplished by use of a triple quadrupole tandem mass spectrometer operated in Multiple Reaction Monitoring (MRM) mode, in which specific precursor to product ion transitions are measured for every analyte and stable isotope labeled internal standard.

Figure 1. Example of a liquid chromatography-tandem mass spectrometry (LC/MS-MS) analysis of bile acids using the Biocrates Bile Acids kit.

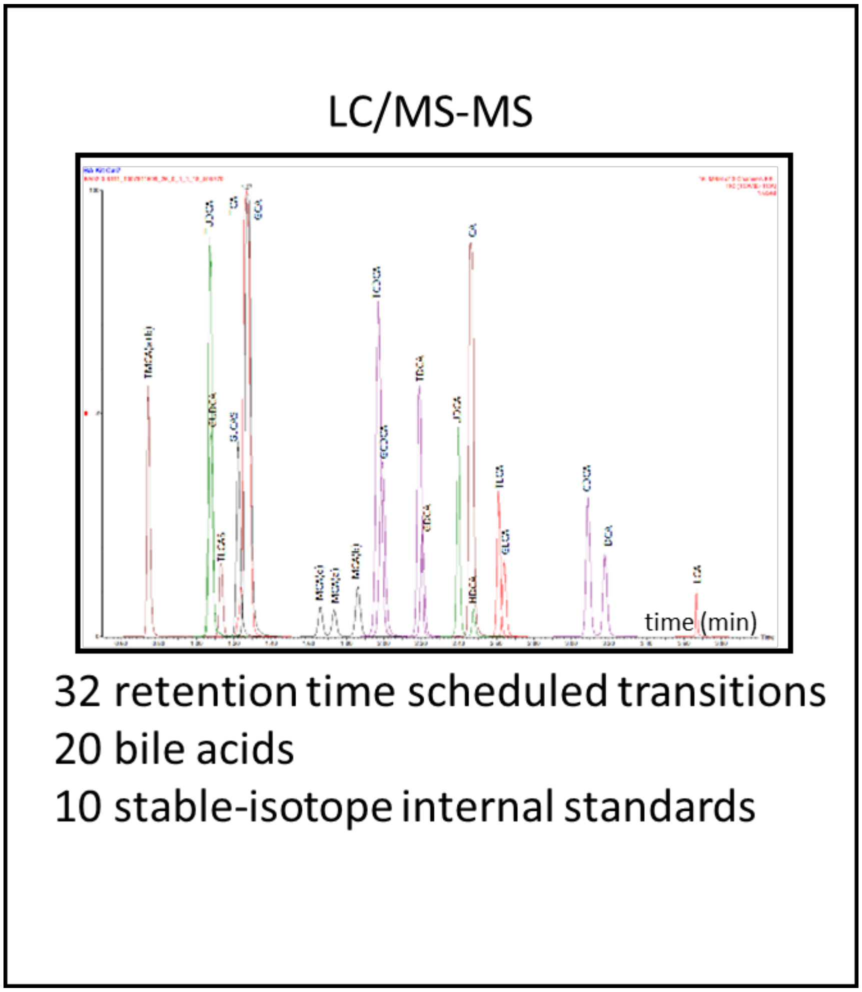

The seven calibration standards provided in the Biocrates Bile Acids kit were used for highly accurate and reproducible quantitation of the bile acids. **Figure 2** shows a schematic and representative example of how the calibration curve was used to back-calculate QC and sample concentrations. Calibration standards were fit with a linear regression using $1/x^2$ weighting.

Figure 2. Schematic depicting the quantitative methodology used in the Biocrates Bile Acids kit.

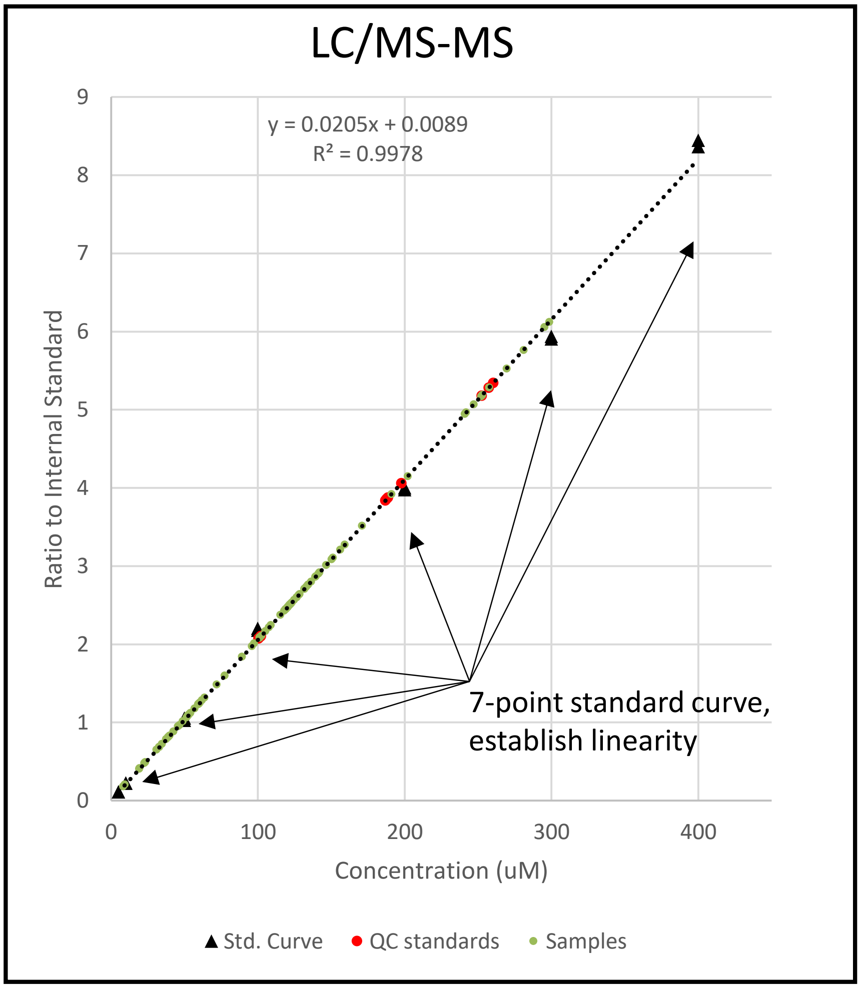

The samples were prepared in a 96-well plate format using the layout shown in **Figure 3**.

Figure 3. Schematic depicting the 96-well plate layout for the analysis of study samples including: blanks, calibration standards, and QC samples from Biocrates. Two additional QC samples were analyzed: the DPMCF Global Reference QC and the sample pool QC (SPQC).

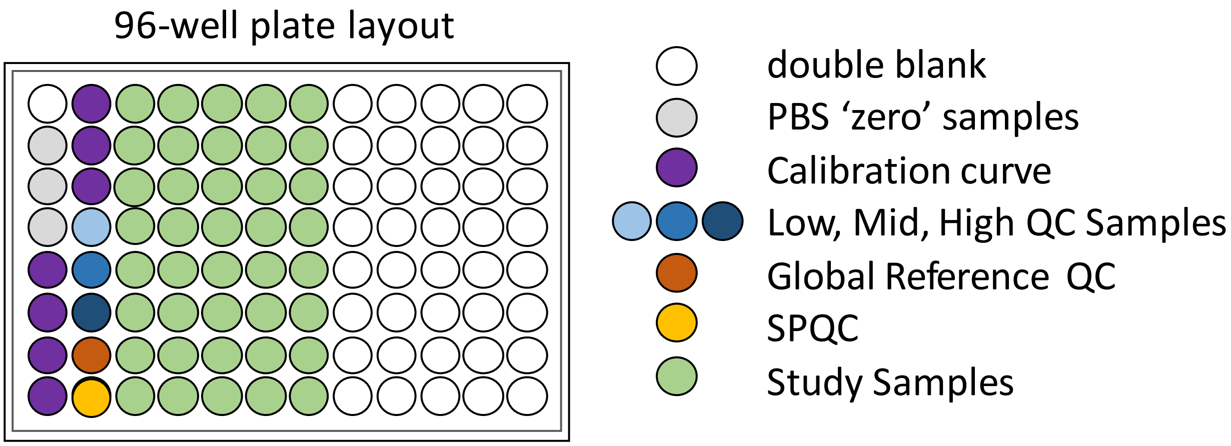

Sample Preparation:

Weighed ileal samples were provided frozen in Precellys soft tissue homogenizing CK14 tubes (Bertin Technologies, Montigny-le-Bretonneux, France). Extraction buffer was prepared by adding 6.8 mL Ethanol to 0.96 mL water and 0.24 mL 100mM pH 7.4 phosphate buffer. Three volumes of extraction buffer were added to each sample 3:1 volume:weight. (For example, 600 µL extraction buffer was added to 200 mg sample.) Samples were then homogenized using three 10-second pulses in the Precellys Evolution between which samples were chilled using the Cryolys cooling system. The samples were then sonicated in an ice water bath for 5 minutes. The homogenized samples were stored at -80C until the day of extraction with the Bile Acids kit.

On the day of bile acids sample extraction the homogenized samples were thawed and vortexed. The samples were then centrifuged at 10000 rcf for 10 minutes in a refrigerated (4C) centrifuge then stored on ice until addition to the bile acids kit plate.

Samples were prepared using the Bile Acids kit (Biocrates Innsbruck, Austria) in strict accordance with their detailed protocol. Addition of 10 µL of the supplied internal standard solution to each well of the 96-well extraction plate was followed by drying under a gentle stream of nitrogen. Ileal samples, blanks, calibration standards, and QCs were added in 10 µL aliquots to the appropriate wells. The plate was then dried under a gentle stream of nitrogen for 10 minutes. The samples were eluted with methanol then diluted with water for UPLC analysis.

A pool of the 40 ileal homogenates was created using equal volumes of all ileal samples. The pooled sample was prepared and analyzed in the same way as the study samples. From the bile acids kit plate this sample was injected once before, once during, and once after the study samples in order to measure the performance of the assay across the sample cohort. The results from these pools can be used to assess potential batch effects. The order of injection of the samples is shown in **Figure 4**.

Figure 4. Schematic depicting the injection order of the samples for UPLC analysis.

Sample Injection Sequence

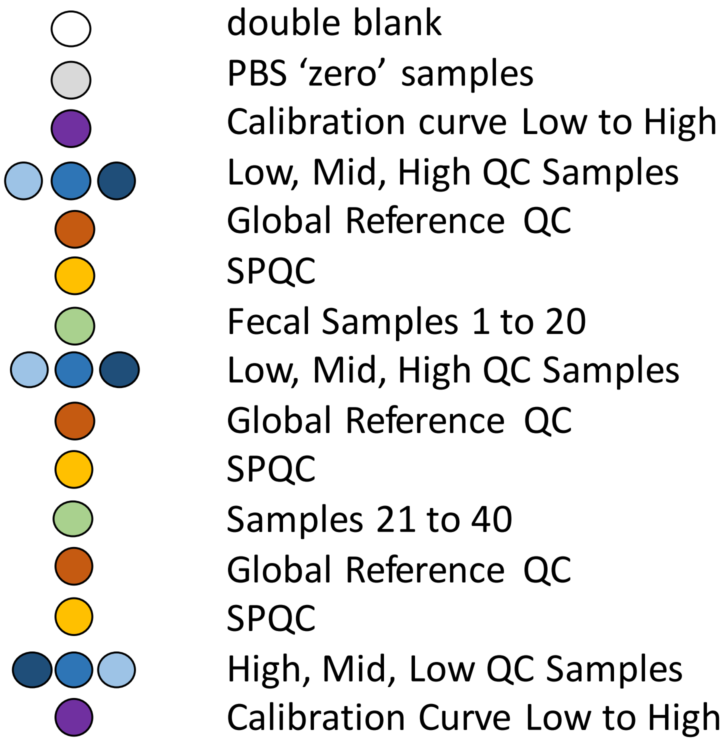

<u>Sample Analysis:</u>

UPLC separation of bile acids was performed using a Waters Acquity UPLC (Milford, MA) with a proprietary reversed-phase UPLC column and guard column provided by Biocrates. Analytes were separated using a gradient from 10mm ammonium acetate, 0.015% formic acid in water to 10mm ammonium acetate, 0.015% formic acid in acetonitrile (65%) and methanol (30%). Total UPLC analysis time was approximately 6 minutes per sample. Using electrospray ionization in negative ion mode, samples were introduced directly into a Xevo TQ-S triple quadrupole mass spectrometer (Waters) operating in the Multiple Reaction Monitoring (MRM) mode. MRM transitions (compound-specific precursor to product ion transitions) for each analyte and internal standard were collected over the appropriate retention time. The UPLC-MS/MS data were imported into Waters application TargetLynx™ for peak integration, calibration, and concentration calculations. The UPLC-MS/MS data from TargetLynx™ were analyzed using Biocrates Met*IDQ*™ software.

<u>Data Return Document Descriptions</u>

A number of documents have been added to the Express Data Repository for Project 5496 including this sample analysis summary. A link to the repository is included here. A description of the data spreadsheets follows the link.
https://discovery.genome.duke.edu/express/resources/5496/.

5496 Bile Acids Data.xlsx

This is an Excel workbook containing five worksheets. The first worksheet (5496 Bile Acids Data nmol_g) contains the calculated concentration data for the ileal homogenates normalized to tissue weight (nmol/g) acquired in the study for bile acids, according to Equation 1 below.

$$\frac{nmol}{g} = \frac{uM\ concentration}{tissue\ factor\left(\frac{g}{L}\right)} * 1000 \qquad (1)$$

The barcode number of the plate is listed in column A. The Sample Bar Code number (column B) is assigned to every sample by the Biocrates Met*IDQ*™ software. The Sample Identification Number in column G is the unique sample identifier assigned by the Proteomics and Metabolomics Sample Submission System. Column D lists the Customer Sample Identification information which were listed on every sample tube provided for analysis. The results for the 5496 SPQC are in rows 48 through 50. Row 52 has the %CV for the study pool sample results. It is our recommendation that if the precision of the

pool sample (as measured by the %CV) for a particular analyte is greater than 25%, then the data for that analyte in the study samples should be flagged for removal from the dataset because of imprecision. Additionally, best practice suggests that analytes which have more than 40% missing values in the study samples should be flagged for potential removal from the dataset because of excess missing values. This last point may not be the case in a test-control study, where either test or control subjects/samples may be below the LOD. In this case, replacement of missing values with LOD/2 is recommended.

Worksheet 2 (Tissue Factors) shows the weight of each sample in grams, the volume of extraction buffer added in liters, and the tissue correction factor in grams/liter for each sample.

The third worksheet (5496 Bile Acids Data uM) contains the calculated concentration data (μM) acquired in the study for bile acids. The second row in this worksheet lists the analytes measured and the status for each one. Row 4 lists the lower limit of detection (LOD) for the analytes. The Biocrates-defined lowest calibration standard and highest calibration standard are listed in Rows 5 and 6. The barcode number of the plate on is listed in column A. The Sample Bar Code number (column B) is assigned to every sample by the Biocrates Me*tIDQ*™ software. The Sample Identification Number in column G is the unique sample identifier assigned by the Proteomics and Metabolomics Sample Submission System. Column D lists the Customer Sample Identification information which were listed on every sample tube provided for analysis.

Table 1 below lists the unique plate barcodes assigned by the Me*tIDQ*™ software for the LC/MS-MS analysis.

Table 1. Plate Barcodes

Plate Number	Plate Barcode for UPLC/MS-MS Analysis.
1	1034741866-1

Table 2 below shows the bile acid analytes measured in this kit, the defined lower limit of detection, calibration range (given as lowest calibration standard to highest calibration standard), the number of study samples that each analyte was measured in (above the LOD), the average measured value for each analyte in the 5496 SPQCs and their respective %CVs. If needed, the 5496 SPQC data from each plate could be utilized to control for batch effects during statistical analysis, however since this study was performed on a single plate, this is not applicable.

Bile Acid	Abbreviation	LOD (µM)	Lowest CS (µM)	Highest CS (µM)	Ileal values >LOD (n)	Average SPQC (µM)	%CV SPQC	Ileal values >ULOQ
Cholic acid	CA	0.004	0.03	75	40	489	5.1	39
Chenodeoxycholic acid	CDCA	0.005	0.02	30	40	28.1	1.6	14
Deoxycholic acid	DCA	0.006	0.02	10	40	49.7	1.2	37
Glycocholic acid	GCA	0.003	0.03	75	40	14.4	1.1	
Glycochenodeoxycholic acid	GCDCA	0.01	0.02	20	37	0.381	0.9	
Glycodeoxycholic acid	GDCA	0.01	0.01	10	40	0.430	4.0	
Glycolithocholic acid	GLCA	0.006	0.01	5	8	NA	NA	
Glycoursodeoxycholic acid	GUDCA	0.006	0.01	10	40	0.608	3.7	
Hyodeoxycholic acid	HDCA	0.005	0.01	5	39	20.9	34.1	29
Lithocholic acid	LCA	0.002	0.01	5	33	0.854	9.9	
Muricholic acid, alpha	MCA(a)	0.008	0.005	5	40	104	6.3	38
Muricholic acid, beta	MCA(b)	0.008	0.01	10	40	324	4.1	39
Muricholic acid, omega	MCA(o)	0.007	0.005	5	40	75.7	4.6	33
Taurocholic acid	TCA	0.008	0.02	50	40	225	3.8	33
Taurochenodeoxycholic acid	TCDCA	0.005	0.01	20	40	58.9	4.2	18
Taurodeoxycholic acid	TDCA	0.001	0.01	10	40	58.4	3.1	18
Taurolithocholic acid	TLCA	0.001	0.01	5	40	0.980	8.9	
Tauromuricholic acid (alpha+beta)	TMCA(a+b)	0.001	0.01	10	40	764.3	10.1	39
Tauroursodeoxycholic acid	TUDCA	0.001	0.01	15	40	80.0	4.5	26
Ursodeoxycholic acid	UDCA	0.002	0.02	30	40	40.6	5.3	17

Table 2. Analysis characteristics of the Bile Acids assay, and measured values for the study pool QCs. The value for the SPQC sample represents the biological mean concentration of all samples in the study, and is helpful for assessment of the measured precision for the analyte, and whether or not the sample average was within the calibration range. Red highlighted analytes either had an average measured value (SPQC) which was below the lower limit of detection (<LOD) or were measurable in less than half of the samples. A significant number of samples had analyte concentrations above the upper limit of quantitation (ULOQ) as indicated.

In the data documents **5496 Bile Acids Data.xlsx** and **5496 Bile Acids QC Data.xlsx** the concentration data (µM) are coded as shown below in Table 3 in order to allow presentation of additional information regarding data quality:

Table 3. Key to the Analyte Status Columns Used in the Data Table 5496 Bile Acids Study Sample Data.xlsx

Valid	Calculated concentrations are based on a standard curve of the analyte listed. (Not all analytes are contained in the calibration standards provided by Biocrates. Therefore, results for some anlaytes will be coded as Semi-Quantitative.)
<Lowest CS: Lowest Calibration Standard>Value>LOD	The value is greater than the LOD but less than the Biocrates-defined lowest calibration standard. These values should be considered reliable except for analytes for which the %CV observed for the pooled sample was greater than 25%.
>Highest CS	Greater than the highest calibration standard
<LOD	Less than the Lower Limit of Detection. The LODs were given by Biocrates, and were not determined using FDA guidelines or a Waters TQ-S mass spectrometer.
Internal Standard out of range	The internal standard peak area for this analyte was slightly lower than the normal range. **NOTE**: There was no adverse effect on the data due to the lower level of internal standard detected. (Not all analytes have internal standards.)
No Interception	No peak was detected in the chromatogram at the appropriate retention time for this analyte.

The fourth worksheet in the workbook contains the µM study sample data with the Status columns removed.

The fifth worksheet in the workbook contains a statistical summarization of the µM data from the Biocrates Met*IDQ*™ application. The Analyte Statistics worksheet lists these summary statistics (Min µM, Max µM, Mean µM, Median µM, 25th Percentile µM, 75th Percentile µM, STD µM, MAD µM, Skewness, Kurtosis, CV [%], CVRobust [%]) by metabolite for all of the samples. The number of results (e.g. samples with usable data) included for each statistical calculation is indicated by "n" in Column C.

Study Samples with Calculated Concentrations Greater than the Highest Calibration Standard

In this study some samples had calculated concentration values that were greater than the highest calibration standard for CA, CDCA, DCA, HDCA, MCA(a), MCA(b), MCA(o), TCA, TCDCA, TDCA, TUDCA, and TMCA(a+b). **These samples have status ">Highest CS" in the data spreadsheet on the third worksheet.** The affected extracted samples were diluted by adding 10 µL extract to 190 µL 1:1 (v/v) Methanol:Water and reinjected. Some chromatograms for CA, TCA and TMCA(a+b) were still saturated after dilution. Please note that these results for the listed analytes should be considered semi-quantitative since they are above the linear calibration range for the assay. A table of the samples and analytes with saturated chromatograms and observed concentrations follows in **Table 4**. Note that in the cases where "NA" is shown in Table 4, the measurement for that analyte was within the calibration range and thus does not present an issue with quantification.

DPMSR Sample Identific ation	Customer Sample Identification		CA (µM)	CDCA (µM)	DCA (µM)	HDCA (µM)	MCA (a) (µM)	MCA (b) (µM)	MCA (o) (µM)	TCA (µM)	TCD CA (µM)	TDCA (µM)	TMCA (a+b) (µM)	TUDCA (µM)	UDCA (µM)
		Highest Calibratio n Standard (µM)	75	30	10	5	5	10	5	50	20	10	10	15	30
		Average SPQC	489	28.1	49.7	20.9	103.8	323.7	75.7	224.7	58.9	58.4	764.3	80.0	40.6
52541	CD248		492	NA	41.3	23.3	18.9	97.2	49.5	148	NA	NA	780	74.2	NA
52542	CD249		432	NA	12.1	4.36	4.86	15.6	4.54	231	117	69.4	854	180	NA
52543	CD250		584	NA	31.2	15.1	49.1	169	103	79.8	NA	NA	823	66	NA
52544	CD251		548	NA	34.6	13.5	26.6	107	65.4	201	NA	NA	882	108	NA
52545	CD252		549	41.1	38.7	24.8	51.9	166	99.9	85.1	NA	NA	784	66.5	53.4
52546	CD254		415	34.7	74.4	46.5	68.3	131	27.1	232	68.8	57.7	888	160	49.2
52547	CD255		447	105	101	40.6	77.4	156	25	222	33	21.2	835	101	56.3
52548	CD256		450	44.8	41.3	19	42.8	72.1	15.4	221	148	81.3	888	187	NA
52549	CD257		298	NA	18.2	15.3	61.6	215	34.9	NA	NA	NA	377	NA	NA
52550	CD245		540	45.2	44.2	1.79	201	511	3.47	NA	NA	NA	475	26.9	NA
52551	CD246		547	50.2	37	<LOD	204	577	4.09	NA	NA	NA	467	NA	39.2
52552	CD247		476	63.6	35.5	NA	217	619	4.63	NA	NA	NA	454	33.2	59.8
52553	CD253		479	94.3	83.5	NA	194	523	3.98	159	38.2	14.7	667	83.5	64.7
52554	CD258		495	NA	32.3	NA	37.6	412	51	237	42.2	10.8	819	39.5	55.5
52555	CD259		550	NA	35.5	19.8	110	1016	126	77.6	NA	NA	493	NA	40
52556	CD260		587	NA	15.3	15.3	84.7	949	131	68.4	NA	NA	337	NA	NA
52557	CD306		475	NA	13.6	42.4	323	665	289	70.5	NA	NA	94.8	NA	NA
52558	CD305		444	56.9	78.6	56.3	260	644	105	105	NA	NA	430	NA	117
52559	CD307		497	NA	37.3	10.3	49.5	121	17.6	233	51.4	29.7	801	67.6	NA
52560	CD308		438	NA	17.8	24.8	159	486	158	NA	NA	NA	42.9	NA	NA
52561	CD309		529	62.7	85.9	27.3	112	301	42.1	103	22.5	12.7	448	39.4	102
52562	CD311		516	NA	38.1	28.6	75.7	548	118	119	NA	NA	213	NA	NA
52563	CD313		458	NA	39.2	13.2	35.5	234	36.7	224	59.4	49.3	768	54.3	NA
52564	CD314		362	NA	34.8	NA	18.4	107	8.12	206	90.1	112	732	116	NA
52565	CD315		454	59.5	93.1	28.1	48.4	256	29.6	219	73.1	71.9	734	67.2	46.6

DPMSR Sample Identification	Customer Sample Identification		CA (µM)	CDCA (µM)	DCA (µM)	HDCA (µM)	MCA (a) (µM)	MCA (b) (µM)	MCA (o) (µM)	TCA (µM)	TCD CA (µM)	TDCA (µM)	TMCA (a+b) (µM)	TUDCA (µM)	UDCA (µM)
52566	CD316		106	NA	NA	NA	6.56	53	3.92	216	126	53.2	739	118	NA
52567	CD317		399	34.1	14.3	NA	29.4	228	16	220	122	36.2	742	75.3	51.6
52568	CD318		257	NA	12.7	NA	12.4	87.7	16.3	179	170	158	774	174	NA
52569	Wt265		514	NA	21	34.3	136	472	306	127	NA	NA	325	11.3	NA
52570	Wt266		504	NA	34.1	34	114	331	172	109	NA	NA	780	24.6	52.1
52571	Wt267		170	NA	NA	5.02	45.9	234	209	NA	NA	NA	NA	NA	NA
52572	Wt274		509	NA	82	32.9	239	571	181	60.6	NA	NA	332	NA	63
52573	Wt275		340	35.1	24.2	58.9	127	495	244	NA	NA	NA	49.6	NA	NA
52574	Wt276		436	84.4	123	20.1	284	592	187	138	NA	NA	337	NA	131
52575	Wi286		356	NA	421	89.3	70.1	89.1	33.2	179	184	461	976	224	60.9
52576	Wi287		454	NA	71.8	47.3	34.8	68.2	26.8	211	72.2	178	719	106	NA
52577	Wi288		455	NA	46.9	15	56.7	179	38.7	87.4	12.4	NA	379	23.8	NA
52578	Wi289		410	NA	14.9	13.3	31.5	46.5	12.8	183	209	146	731	151	NA
52579	Wt292		NA	NA	NA	NA	NA	NA	NA	131	184	312	688	169	NA
52580	Wt297		466	NA	57.9	50.4	188	646	179	73.7	NA	NA	681	NA	43.1

Table 4. Samples and Analytes above the Highest Calibration Standard

5496 Bile Acids QC Data.xlsx

This worksheet contains the Biocrates plasma QC concentration data for three levels of QCs. The measurements for the QC samples are provided in order to confirm that quantification of the metabolites across a wide dynamic range performed in this analysis is generally accurate and reproducible. The QC values showed excellent reproducibility (Table 3).

Table 3. Reproducibility of QC Sample Analyses

QC Level	Average % CV	Min %CV	Max %CV
Low	7.6	1.0	28.9
Medium	2.5	0.9	8.8
High	2.5	0.4	8.4

The DPMCF Global Reference QC results are used internally by the Core to assess the reproducibility of the assay within and across projects.

5496 Bile Acids Data Dictionary Column Header Definitions

Excel workbook 5496 Data Dictionary contains useful information about the analytes detected using the Biocrates Bile Acids kit.

1) FLDNAME
2) DESCRIPTION
3) MOLECULARWEIGHT
4) MOLECULARFORMULA
5) KEGG
    a. Kyoto Encyclopedia of Genes and Genomes
    b. http://www.genome.jp/kegg/
6) MOLECULE
    a. Alternate name
7) CAS
    a. Chemical Abstracts Service Registry Number
    b. https://www.cas.org/content/chemical-substances
8) INCHIKEY
    a. IUPAC International Chemical Identifier Key
    b. http://www.iupac.org/home/publications/e-resources/inchi.html
9) HMDB
    a. Human Metabolome Database
    b. http://www.hmdb.ca/
10) CHEBI
    a. Chemical Entities of Biological Interest
    b. https://www.ebi.ac.uk/chebi/
11) IUPAC
    a. International Union of Pure and Applied Chemistry
    b. http://www.iupac.org/
12) ISOMERIC
    a. SMILES (simplified molecular-input line-entry system)
    b. http://en.wikipedia.org/wiki/Simplified_molecular-input_line-entry_system
13) INCHI
    a. IUPAC International Chemical Identifier
    b. http://www.iupac.org/home/publications/e-resources/inchi.html
14) MESH

   a. NCBI Medical Subject Headings name
   b. http://www.ncbi.nlm.nih.gov/mesh

15) LMID
   a. Lipid Maps
   b. http://www.lipidmaps.org/

Principal Components Analysis (PCA) Plots

In order to assess general variability of the sample data and to look for sample outliers; a Principal Components Analysis (PCA) was performed for bile acids using JMP® Pro v14.0 software (SAS, Cary, NC) from table **5496 Bile Acids Data.xlsx**. Analytes with measurable concentrations for more than half of the samples were included in the PCA analysis. For missing values within the remaining analytes, these missing values were replaced with the Biocrates LOD value for that analyte. The restricted maximum likelihood (REML) method was used for correlation during the PCA analysis in JMP Pro.

Figure 4 below shows the PCA plot performed in JMP using the values in the "*Status Columns Removed uM"* tab from **5496 Bile Acids Data.xlsx.** Note in this analysis that separate colors were assigned to study samples (red for CD and blue for WT) and SPQC (green) in order to evaluate variability and to check for outliers. The results for the pool (blue dots) are clustered together centrally within the samples from which they were created which implies that very minimal technical variability

Recommended Steps Before Statistical Analysis

- Remove analytes with >40% missing values (may supplement with test for statistical difference in proportion of missing values between groups, if unblinded)
- Replace missing values ("<LOD") with either the Biocrates LOD value or (LOD)/2.
- Scale log2 as necessary for analytes with non-normal distributions. This is likely to be needed for bile acids data.

Figure 4. Principal Components Analysis of Bile Acids μM concentration Data

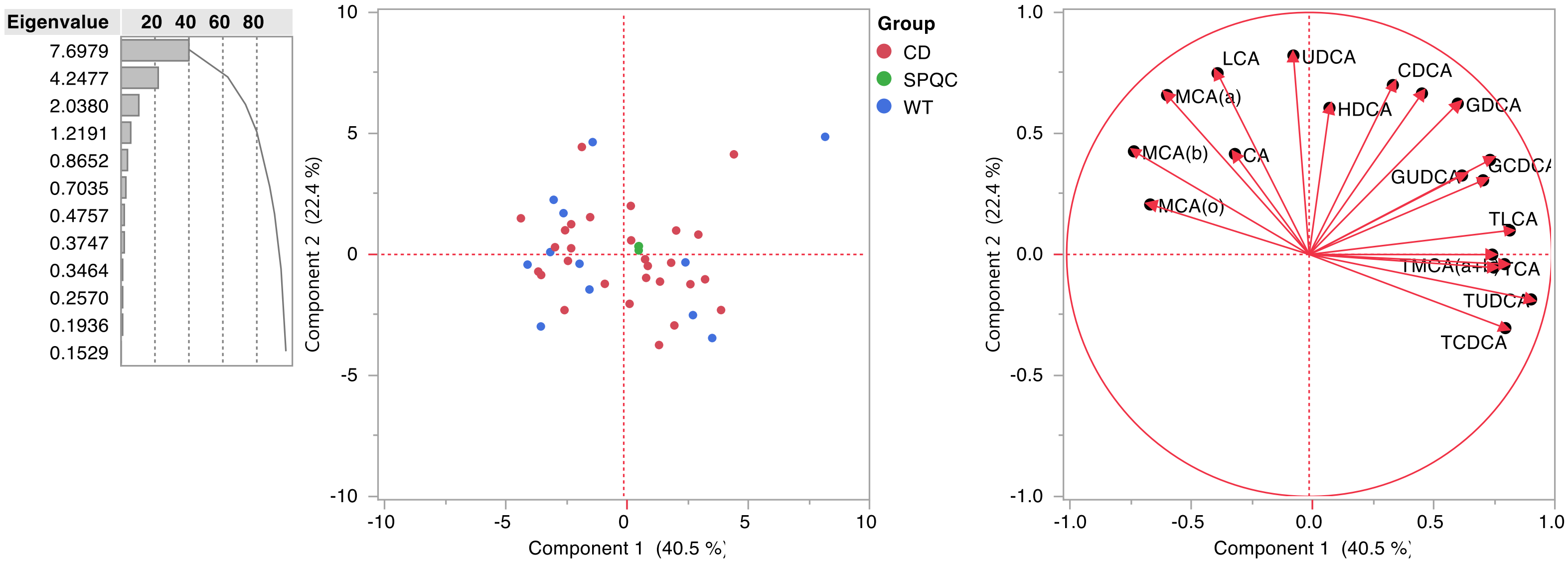

DPMSR Project 5496
