# Supplementary material for: Defective humoral immunity disrupts bile acid homeostasis which promotes inflammatory disease of the small bowel
Source: Nat Commun. 2022 Jan 26;13:525. doi: 10.1038/s41467-022-28126-w (PMC8792037; doi:10.1038/s41467-022-28126-w)
Supplement: Supplementary file 2 — Description of Additional Supplementary Files [file 41467_2022_28126_MOESM2_ESM.docx]

**Description of Additional Supplementary Files**

**Filename:** Supplementary Data 1

**Description:** A table providing raw data from UPLC-MS analysis of bile acid concentrations that was used to create Figures 1D-G, 7D and 7E is provided in this file.

**Filename:** Supplementary Data 2

**Description:** Rarified OTU tables used to generate 16S datasets are provided in this file. Relevant to Figure 1 and Figure 5.

**Filename:** Supplementary Data 3

**Description:** A table listing the differentially abundant OTUs as inferred from DS-FDR analysis between WT and CD19^-/-^ mice is provided in this file. Relevant to Figure 1.

**Filename:** Supplementary Data 4

**Description:** A table summarizing the abundance of ileal transcript reads and results of statistical analyses from genes associated with BA metabolism between WT and CD19^-/-^ mice is provided. Relevant to Figure 2.

**Filename:** Supplementary Data 5

**Description:** A table summarizing the abundance of liver transcript reads and statistical analyses for differentially enriched genes from the liver of WT and CD19^-/-^ mice is provided. Relevant to Figure 2.

**Filename:** Supplementary Data 6

**Description:** A table summarizing the abundance of liver transcript reads and results of statistical analyses from genes associated with BA metabolism between WT and CD19^-/-^ mice is provided. Relevant to Figure 2.
