# Supplementary material for: Defective humoral immunity disrupts bile acid homeostasis which promotes inflammatory disease of the small bowel
Source: Nat Commun. 2022 Jan 26;13:525. doi: 10.1038/s41467-022-28126-w (PMC8792037; doi:10.1038/s41467-022-28126-w)
Supplement: Supplementary file 9 — Reporting Summary [file 41467_2022_28126_MOESM9_ESM.pdf]

nature research

Corresponding author(s): Jason L. Kubinak

Last updated by author(s): 2021/11/18

# Reporting Summary

Nature Research wishes to improve the reproducibility of the work that we publish. This form provides structure for consistency and transparency in reporting. For further information on Nature Research policies, seeAuthors & Referees and theEditorial Policy Checklist .

## Statistics

For all statistical analyses, confirm that the following items are present in the figure legend, table legend, main text, or Methods section.

| n/a | Confirmed | |
|---|---|---|
| ☐ | ☒ | The exact sample size (*n*) for each experimental group/condition, given as a discrete number and unit of measurement |
| ☐ | ☒ | A statement on whether measurements were taken from distinct samples or whether the same sample was measured repeatedly |
| ☐ | ☒ | The statistical test(s) used AND whether they are one- or two-sided<br>*Only common tests should be described solely by name; describe more complex techniques in the Methods section.* |
| ☒ | ☐ | A description of all covariates tested |
| ☐ | ☒ | A description of any assumptions or corrections, such as tests of normality and adjustment for multiple comparisons |
| ☐ | ☒ | A full description of the statistical parameters including central tendency (e.g. means) or other basic estimates (e.g. regression coefficient) AND variation (e.g. standard deviation) or associated estimates of uncertainty (e.g. confidence intervals) |
| ☐ | ☒ | For null hypothesis testing, the test statistic (e.g. *F*, *t*, *r*) with confidence intervals, effect sizes, degrees of freedom and *P* value noted<br>*Give P values as exact values whenever suitable.* |
| ☒ | ☐ | For Bayesian analysis, information on the choice of priors and Markov chain Monte Carlo settings |
| ☒ | ☐ | For hierarchical and complex designs, identification of the appropriate level for tests and full reporting of outcomes |
| ☐ | ☒ | Estimates of effect sizes (e.g. Cohen's *d*, Pearson's *r*), indicating how they were calculated |

*Our web collection on statistics for biologists contains articles on many of the points above.*

## Software and code

Policy information about availability of computer code

| | |
|---|---|
| Data collection | no custom code was used to obtain data in this study. Flow Cytometry: FACS Diva 8.0.1. RNAseq: Real-Time Analysis (RTA) software v 2.4.11 and System Suite v 2.0. (Illumina). UPLC-MS: UPLC-MS/MS data were imported into Waters application TargetLynx™ for peak integration, calibration, and concentration calculations. The UPLC-MS/MS data from TargetLynx™ were analyzed using Biocrates MetIDQ™ software. |
| Data analysis | Basic univariate and bi-variate analyses were conducted using PRISM8.0 software. R-studio was utilized to perform gene enrichment analyses for RNAseq experiments. The QIIME2.0 analysis pipeline was utilized for 16S rRNA gene sequencing analyses. For RNAseq datasets, sequences were aligned to the Mus Musculus genome GRCm38.p5 (GCA_000001635.7, ensemble release-88) using STAR v2.4. Samtools (v1.2) was used to convert aligned sam files to bam files and reads were counted using the featureCounts function of the Subreads package with Gencode.vM19.basic.annotation.gtf annotation file. Differential expression analysis was performed in R using the edgeR package. Raw counts were normalized using the Trimmed Mean of M-values (TMM) method. The normalized read counts were fitted to a quasi-likelihood negative binomial generalized log-linear model using the function glmQLFit. Genewise statistical tests for significant differential expression were conducted with empirical Bayes quasi-likelihood F-tests using the function glmQLFTest. |

For manuscripts utilizing custom algorithms or software that are central to the research but not yet described in published literature, software must be made available to editors/reviewers. We strongly encourage code deposition in a community repository (e.g. GitHub). See the Nature Research guidelines for submitting code & software for further information.

## Data

Policy information about availability of data

All manuscripts must include a data availability statement. This statement should provide the following information, where applicable:

- Accession codes, unique identifiers, or web links for publicly available datasets
- A list of figures that have associated raw data
- A description of any restrictions on data availability

Source data are provided with this paper. Supplementary Tables T1-T6 that are referenced in the main document are provided as supplementary source data alongside the published paper (filename 'Bcells_and_BileAcids_Source_Data'). Supplementary Tables T7 and T8 have been provided within the Supplemental File associated with this paper. Source data for datasets summarized in this manuscript have been deposited and made publicly available through the Dryad Data Repository (https://doi.org/10.5061/dryad.rxwdbrv9h). Raw sequence data and relevant metadata for all 16S analyses shown in this manuscript have been deposited in the NCBI short read archive (SRA) under Bioproject ID#PRJNA773874. Raw sequence data and relevant metadata for ileal and liver RNAseq datasets have been deposited into the NCBI Gene Expression Omnibus (GEO) under the GEO identifiers GSE186435 and GSE186436, respectively.

# Field-specific reporting

Please select the one below that is the best fit for your research. If you are not sure, read the appropriate sections before making your selection.

- [x] Life sciences
- [ ] Behavioural & social sciences
- [ ] Ecological, evolutionary & environmental sciences

For a reference copy of the document with all sections, see nature.com/documents/nr-reporting-summary-flat.pdf

# Life sciences study design

All studies must disclose on these points even when the disclosure is negative.

| | |
|---|---|
| Sample size | Power analyses were not performed to pre-determine experimental group sizes for animal studies. Instead, appropriate group sizes were estimated based on prior effect sizes observed for similar models in a previous study by our group (PUBMED#31708923) as well as animal availability. Animal numbers are provided throughout in the figure legends. |
| Data exclusions | Data exclusion was pre-established in all experiments and based on statistical criteria. Specifically, using the ROUT method of outlier identification, a false discovery rate of 0.1 was applied to relevant data-sets. This q-value results in a 99% likelihood of correctly identifying statistical outliers and this was the maximum q-value threshold applied to all data-sets. Outlier analysis was performed on data-sets shown in Figure 1A, 1B, and 6C using Prism8.0 software. |
| Replication | 2-3 replicate experiments were conducted for all datasets shown. Datasets represent pooled data across replicates. All attempts at replication were successful. |
| Randomization | For adoptive transfer experiments and mono-colonization studies of CD19 littermates, male and female mice of appropriate ages were randomly assigned to experimental groups. Randomization involved allocating mice derived from different litters equally between experimental treatments. |
| Blinding | For pathology scoring, our Pathologist (Dr. Ioulia Chatzistamou) was blinded to animal treatment group. Blinding was not relevant to any other aspect of our study because those involved in data collection and analysis (the first- and senior-author) designed all experiments and needed to know the specific genotype/treatment. |

# Reporting for specific materials, systems and methods

We require information from authors about some types of materials, experimental systems and methods used in many studies. Here, indicate whether each material, system or method listed is relevant to your study. If you are not sure if a list item applies to your research, read the appropriate section before selecting a response.

## Materials & experimental systems

| n/a | Involved in the study | |
|---|---|---|
| [ ] | [x] | Antibodies |
| [x] | [ ] | Eukaryotic cell lines |
| [x] | [ ] | Palaeontology |
| [ ] | [x] | Animals and other organisms |
| [x] | [ ] | Human research participants |
| [x] | [ ] | Clinical data |

## Methods

| n/a | Involved in the study | |
|---|---|---|
| [x] | [ ] | ChIP-seq |
| [ ] | [x] | Flow cytometry |
| [x] | [ ] | MRI-based neuroimaging |

## Antibodies

| | |
|---|---|
| Antibodies used | Anti-mouse CD45, APC.Cy7 conjugated, clone 30-F11, BioLegend, cat#103113<br>Anti-mouse CD45, APC conjugated, clone 30-F11, BioLegend, cat#103115<br>Anti-mouse CD326, Alexa488 conjugated, clone G8.8, BioLegend, cat#118210<br>Anti-mouse CD326, PE.Cy7 conjugated, clone G8.8, BioLegend, cat#118215<br>Anti-mouse Ki-67, APC conjugated, clone 16A8, BioLegend, cat#652405<br>Annexin V, APC, BioLegend, cat#640920<br>Propidium Iodide, PE, BioLegend, cat#421301<br>Zombie Green, FITC, BioLegend, cat#423111<br>MitoSpy NIR DiIC1(5), APC, BioLegend, cat#424807 |
| Validation | All antibodies and associated reagents listed above were purchased from Biolegend. Biolegend performs routine quality control testing to minimize lot-to-lot variation in product performance. QC validation of antibodies is outlined on the vendor website as follows: "Each lot is compared to an internally established "gold standard" to maintain lot-to-lot consistency. We also conduct wide-scale stability studies to guarantee an accurate shelf-life for our products. Additionally, we test the majority of our products on endogenous cells rather than transfected or immortal cells that may overexpress the analyte." For flow cytometry reagents, the following validations are performed: (1) Specificity testing of 1-3 target cell types with either single- or multi-color analysis (including positive and negative cell types), (2) Once specificity is confirmed, each new lot must perform with similar intensity to the in-date reference lot,. Brightness (MFI) is evaluated from both positive and negative populations, and (3) Each lot product is validated by QC testing with a series of titration dilutions. |

## Animals and other organisms

Policy information about studies involving animals; ARRIVE guidelines recommended for reporting animal research

| | |
|---|---|
| Laboratory animals | Mus musculus, WTC57BL/6 and congenic CD19-/- mice, male and female mice, 8-16 weeks old |
| Wild animals | no wild animals were used in this study |
| Field-collected samples | no field-collected samples were used in this study |
| Ethics oversight | University of South Carolina Institutional Animal Care and Use Committee (Protocol #101292) |

Note that full information on the approval of the study protocol must also be provided in the manuscript.

## Flow Cytometry

### Plots

Confirm that:

- [x] The axis labels state the marker and fluorochrome used (e.g. CD4-FITC).
- [x] The axis scales are clearly visible. Include numbers along axes only for bottom left plot of group (a 'group' is an analysis of identical markers).
- [x] All plots are contour plots with outliers or pseudocolor plots.
- [x] A numerical value for number of cells or percentage (with statistics) is provided.

### Methodology

| | |
|---|---|
| Sample preparation | A detailed description of sample processing for flow cytometry experiments are provided in the methods section of our manuscript. |
| Instrument | A BD FACS ARIA II Cell Sorter and BD Accuri C6 Flow Cytometer were used for flow cytometry analyses shown in this study |
| Software | BD FACS Diva was used for data collection and FLOJO9.0 software was used for data analysis and plot construction |
| Cell population abundance | The STEMCELL Pan-B Cell Isolation Kit was used for isolating splenic B cell subsets from WT and CD19-/- mice, which purifies cells to ~98% purity. Two sequential cell purifications were performed on isolated splenocytes to ensure a high purity of B cells. |
| Gating strategy | Gating rubric for enumerating apoptotic IECs in cell isolations derived from SI (ileal) tissue. SI cells from the ileums of mice were isolated as described in methods section. Cells were stained with anti-mouse CD45, anti-mouse EpCAM, PI, and Annexin V. We first gated on single cells, and then gated on the CD45- population. From the CD45- population we then gated on EpCAM+PI- events (living IECs). The proportion of healthy (AnnV-) versus apoptotic cells (ie. AnnV+) were then gated. The same gating strategy was used for all IEC apoptosis experiments described in this manuscript. |

- [x] Tick this box to confirm that a figure exemplifying the gating strategy is provided in the Supplementary Information.
